# The widespread *Leodamas chevalieri* (Annelida, Orbiniidae) revealed as a species complex, with descriptions of a new species and a new record for the Arabian region

**DOI:** 10.3897/zookeys.1276.173213

**Published:** 2026-04-03

**Authors:** Miguel A. Meca, Anna Zhadan, Manal Al-Kandari, Nataliya Budaeva

**Affiliations:** 1 Department of Natural History, University Museum of Bergen, University of Bergen, Allégaten 41, 5007, Bergen, Norway N.A. Pertsov White Sea Biological Station, Biological Faculty, M.V. Lomonosov Moscow State University Moscow Russia; 2 Centre d’Estudis Avançats de Blanes (CEAB-CSIC), Carrer d’Accés a la Cala Sant Francesc 14, 17300 Blanes (Girona), Catalunya, Spain Centre d’Estudis Avançats de Blanes (CEAB-CSIC) Blanes Spain; 3 N.A. Pertsov White Sea Biological Station, Biological Faculty, M.V. Lomonosov Moscow State University, Leninskie Gory 1–12, Moscow 119234, Russia Department of Natural History, University Museum of Bergen, University of Bergen Bergen Norway; 4 Coastal and Marine Research Program, Environment and Life Sciences Research Center, Kuwait Institute for Scientific Research, P. O. Box 1638, Salmiya 22017, Kuwait Coastal and Marine Research Program, Environment and Life Sciences Research Center, Kuwait Institute for Scientific Research Salmiya Kuwait

**Keywords:** Arabia, COI, cosmopolitan, integrative taxonomy, ITS2, *Leodamas chevalieri*, SEM, species complex

## Abstract

*Leodamas chevalieri*, the only *Leodamas* species reported in the Arabian region, was originally described from Senegal, western Africa. In this work, the morphological and genetic (COI and ITS2) variation of several populations of *Leodamas* from different localities in Kuwait, Oman, and western Africa were analyzed. The type material of *L. chevalieri* is described, and molecular data provided for specimens collected close to the type locality. Based on the molecular analysis, at least twelve lineages were recognized in the studied area (eight in the Arabian region and four in the western Africa), with *L. chevalieri* being a species complex composed of at least eleven lineages. The species name *Leodamas chevalieri* is assigned to the lineage found close to the type locality, restricting the species distribution to the coastal areas of Senegal. One lineage from off Kuwait was described as a new species, *Leodamas edgari***sp. nov**., differing from *L. chevalieri* in fine chaetal characters. Another lineage was identified as *Leodamas gracilis*, originally described from Sri Lanka, representing a new record of this species for the Arabian region. Further investigation is needed to describe or to name the other lineages belonging to the *L. chevalieri* species complex. With the description of *L. edgari***sp. nov**., the genus comprises 31 species worldwide. However, the number of discovered genetic lineages in this study indicates a high proportion of undescribed species diversity in the genus.

## Introduction

The concept of “cosmopolitan” species in marine annelids was widespread until the 1980s, when the taxonomic efforts increased significantly and became more collaborative, with gradual technological advancement which allowed for greater morphological detail (Scanning Electron Microscopy, or SEM, and digital photography), easier access to bibliographic and type material (internet era), and the implementation of molecular methods (Hutchings and Kupriyanova 2018). These factors enabled us to question the cosmopolitan status of many species and, although most were revealed to be complexes of morphologically close species, real cosmopolitan species exist, but mainly in the deep sea (Meißner et al. 2023; Budaeva et al. 2024) or as fouling species. The latter is reported for a cosmopolitan orbiniid, *Proscoloplos cygnochaetus* Day, 1954, occurring in the temperate waters in the whole southern hemisphere and showing no genetic structuration among populations (Meyer et al. 2008). Most probably, its very wide distribution is facilitated by human transport by vessels.

Orbiniidae systematics is characterized by a lack of congruence between morphological and molecular data, with the genera *Scoloplos*, *Leitoscoloplos*, *Leodamas*, *Orbinia*, and *Phylo* recovered as paraphyletic in the two main molecular works on Orbiniidae performed by Bleidorn et al. (2009) and Zhadan et al. (2015). Meca (2025) conducted a phylogenetic study based on genome skimming and robust taxon coverage which will lead to redefining some genera and, consequently, changes in their species composition. Regarding *Leodamas*, we provide here an emended diagnosis and reorganize the number of species based on Meca’s (2025) results (see remarks on subsection of *Leodamas* diagnosis for further details). In Meca et al. (2021: Suppl. material 1: table SS2), 32 species following Blake’s diagnoses in Blake (2017, 2020, 2021) were listed; however, in the present study we consider 30 species belonging to *Leodamas* according to our emended diagnosis.

*Leodamas* Kinberg, 1866 is currently the third most species-rich genus in the family Orbiniidae with, as mentioned, 30 described valid species, commonly found on muddy or sandy shores from intertidal to approximately 300 m deep (Meca et al. 2021). Species of *Leodamas* can be recognized by a conical prostomium, clear thorax-abdomen distinction, one achaetous peristomial ring, branchiae starting in anterior segments, thoracic neuropodia bearing large and numerous uncini sometimes accompanied by a few crenulated capillaries, thoracic neuropodial postchaetal lobes usually as ridges without prominent lobes, abdominal neuropodia with a single robust protruding acicula, and abdominal notopodia usually bearing forked chaetae. *Leodamas* has been recently revised by Blake (2017) based on morphological data. Sun et al. (2018) provided an identification key for all *Leodamas* species. However, the absence of a complete revision of the genus using an integrative approach is a pressing issue that needs to be addressed.

*Leodamas chevalieri* (Fauvel, 1902) is the only recorded species of the genus in the Arabian region, including the Red Sea, the Gulf of Aden, the Arabian Gulf, and the Oman coast (Wehe and Fiege 2002). This species was originally described from the estuary of the Casamance River (Senegal, West Africa) and since then has been widely reported from off Morocco, the Red Sea, the Gulf of Aden, the Arabian Gulf, India, and Malaysia (Gravier 1906; Fauvel 1919, 1953; Wesenberg-Lund 1949; Amoureux 1976; Ong 1995; Blake 2017; Al-Kandari et al. 2019), from muddy sandy shores to ~30 m deep. Additionally, one subspecies, *Leodamas chevalieri candiensis* (Harmelin, 1969), was described from Crete (Mediterranean Sea) from seagrass habitats (*Cymodocea nodosa* and *Halophila stipulacea*) at 7–11 m. Several species were described from the Western Indo-Pacific among which was *Leodamas gracilis* (Pillai, 1961) from Tambalagam Bay (Sri Lanka).

The aim of the present study is to analyze morphological variation, genetic divergence, and phylogenetic relationships among several intertidal populations of *Leodamas* from different localities in Kuwait and Oman. To confirm the presence of this species in the Arabian region, we compared the Arabian populations with that from Senegal, the type locality of *L. chevalieri*. We further redescribed the type material of *L. chevalieri* and designated the lectotype for a more precise definition of the morphological characteristics of the species.

## Material and methods

### Material collection

In West Africa, the specimens were collected during the R/V Dr Fridtjof Nansen cruise as a part of the Canary Current Large Marine Ecosystem (CCLME) project in 2011. The sediment samples were obtained using an epibenthic sledge and sieved onboard. In Kuwait, the specimens were collected during the autumn, winter, and spring seasons from 2014–2020 in the intertidal zone by shoveling or using 25 × 25 cm square metal box corer, 15 cm in depth. In West Africa and Kuwait, the specimens were preserved directly in 96% ethanol or fixed first in 10% buffered formalin solution and later transferred into 75% ethanol. In Oman, the specimens were sampled during the BioBlitz survey in coastal areas in February 2022. The samples were collected in the intertidal and subtidal zones by shoveling or scuba diving. The sediment was sieved through a 300–1000-µm sieve. Worms were picked out and anesthetized in an isotonic MgCl_2_ solution for 30 min, photographed live and later preserved in 10% buffered formalin solution with a separate tissue sample fixed in 96% ethanol. Formalin-fixed specimens were later transferred into 75% ethanol (Suppl. material 1: table SS1). The specimens are deposited in the Zoological Museum of Moscow State University (White Sea Branch), Russia (**ZMMU WS**), the Florida Museum of Natural History, University of Florida, Gainesville, Florida, USA (**FLMNH**), the Kuwait Institute of Scientific Research, Kuwait City, Kuwait (**KISR**), and the Invertebrate collection of the Department of Natural History, University Museum of Bergen, University of Bergen, Norway (**ZMBN**). The syntypes of *Leodamas chevalieri* were borrowed for examination from the National Museum of Natural History, Paris, France (**MNHN**).

### Morphological methods

Preserved specimens were examined under a Leica M125 stereomicroscope, and glycerol mounts of parapodia were studied using a Leica AS compound microscope. Photographs were taken with a digital camera Leica DMC5400 mounted on a Leica M205C stereomicroscope and with a digital camera Leica DMC4500 mounted on a Leica DM6000 B compound microscope, both equipped with the Z-stack function on Leica LAS software. The images were edited in Adobe Photoshop 2021 22.3.1. An aqueous solution of methylene blue was used to add contrast to external structures, such as branchiae, papillae, and parapodia, and to highlight segmental borders. For SEM, specimens were dehydrated in a graded ethanol series (20–25 min per step), then transferred to acetone and critical point dried. Whole specimens and their fragments were coated with platinum-palladium alloy and examined using the following scanning electron microscopes: Camscan S-2 (Cambridge Instruments, London, United Kingdom) and JEOL JSM-6380LA (JEOL Ltd., Tokyo, Japan) at the Laboratory of Electron Microscopy at the Biological Faculty of Moscow University (Moscow, Russia); Neoscope JCM-7000 (JEOL Ltd., Tokio, Japan) at the White Sea Biological station (Biological Faculty of Moscow State University, Russia); and Hitachi SU3500 (Hitachi Ltd., Tokyo, Japan) at the service of Electron Microscopy and Microanalysis of MNHN (National Museum of Natural History, Paris, France).

### DNA extraction, PCR amplification, and sequencing

Genomic DNA was extracted using QuickExtract^TM^ DNA Extraction Solution. A small piece of tissue, usually three or four segments, was placed in 70 μl of QuickExtract solution, and incubated at 65 °C for 45 min followed by 2 min at 95 °C in a dry block thermostat. A fragment of one mitochondrial (COI) and one nuclear (ITS2 with a flanking region of 28S) marker was amplified using the following primers respectively: PolyLCO, 5’-GAYTATWTTCAACAAATCATAAAGATATTGG-3’ and PolyHCO, 5’- TAMACTTCWGGGTGACCAAARAATCA-3’ (Carr et al. 2011); ITS5.8SFPOLY, 5’-GAATTGCAGGACACATTGAAC-3’ (Nygren et al. 2009) and 28SC2-R, 5’-TGAACTCTCTCTTCAAAGTTCTTTTC-3’ (Vân Le et al. 1993). The total volume of PCR reactions was 25 μl containing: 17.35 μl of nuclease-free water, 2.5 μl of 10× buffer, 2 μl of nucleotide mix (2.5 mM each dNTP), 1 μl of each primer (10 μM), 0.15 μl of TAQ DNA polymerase (Clontech, concentration of 5 U/μl), 1 μl of DNA template. PCR temperature profile was 94 °C for 5 min, 5 cycles x (94 °C for 40 s + 45 °C for 40 s + 72 °C for 2 min), 40 cycles x (94 °C for 40 s + 48 °C for 40 s + 72 °C for 2 s) and 72 °C for 7 min (COI) and 96 °C for 4 min, 45 cycles x (94 °C for 30 s + 48 °C for 30 s + 72 °C for 1 min) and 72 °C for 8 min (ITS2). Amplified PCR products were analyzed by electrophoresis on a 1% agarose gel stained with GelRed Nucleic Acid Stain and then sent to Macrogen Inc. facilities (Amsterdam, the Netherlands) for purification and bidirectional sequencing. Consensus sequences were generated and edited in Geneious Prime 2021.2.2 (Biomatters Ltd., Auckland, New Zealand; Kearse et al. 2012).

### Phylogenetic analyses

Forty-three COI and 37 ITS2 sequences were generated in the present study. Three more COI sequences of *Leodamas* were obtained from GenBank. *Orbiniella griegi* Meca & Budaeva, 2024 was used as an outgroup (Suppl. material 1: table SS1). The dataset of each marker was aligned individually using MAFFT v. 7.450 (Katoh and Standley 2013) implemented in Geneious Prime under the L–INS-i algorithm and default parameters. Both alignments were concatenated into a single matrix in Geneious Prime. Best-fit models for each partition were selected using the Akaike Information Criterion (AIC) in PartitionFinder v. 2.1.1 (Lanfear et al. 2017). We applied the General Time-Reversible model with an estimated proportion of invariant sites and gamma distributed rates across sites (GTR+I+G) for the ITS2 and the second and third positions of COI, and the GTR model with gamma distributed rates across sites (GTR+G) for the first position of COI. Phylogenetic analyses were conducted for individual markers and for the concatenated matrix using CIPRES Science Gateway 3.3 (Miller et al. 2012). Maximum likelihood (ML) analyses were conducted using IQ-TREE v. 2.1.2 (Nguyen et al. 2015) with 1000 bootstrap replicates. Bayesian inference (BI) analyses were done using MrBayes v. 3.2.7 (Ronquist et al. 2012) with two independent runs (each performed for eight Markov Chain Monte Carlo simulations) for 40 million generations for the single marker datasets and for 100 million for the combined dataset, sampled every 1000 generations and 25% of the initial trees discarded as burn-in. We considered the runs converged and the results of the analyses accepted with Average Standard Deviation of Split Frequencies (ASDSF) < 0.03 and effective sample size of parameters (ESS > 200) estimated in Tracer 1.7.1 (Rambaut et al. 2018). The concatenated BI tree was edited in CorelDRAW X7, whilst the rest of the trees were edited with Inskape v. 1.1 (https://inkscape.org/).

### Species delimitation and genetic distances

To delineate putative species in our datasets, we used the Poisson Tree Processes model (PTP) (Zhang et al. 2013) and the Assemble Species by Automatic Partitioning (ASAP) (Puillandre et al. 2021) for individual markers. PTP was inferred through its web server (https://species.h-its.org/), using the obtained BI rooted trees with pruned outgroups for COI and ITS2, 100000 generations and default settings. The convergence of MCMC runs was checked in the maximum-likelihood plot generated by the software. The ASAP was applied through its web server (https://bioinfo.mnhn.fr/abi/public/asap/) using alignment files and a p-distance model. Between and within groups average uncorrected p-distances (with gaps treated as pairwise deletion) were calculated in MEGAX v. 10.2.4 (Kumar et al. 2018) for all putative species. Additionally, uncorrected p-distances in 16S were calculated between the specimens belonging to what is hereafter referred to as Lineage 15 (using two sequences extracted from genome skimming data of a phylogenetic study of Orbiniidae (Meca 2025), *L. australiensis* (Hartmann-Schröder, 1979) from an Australian population (two sequences obtained from GenBank), and *L. australiensis* from a Mediterranean population (one sequence kindly provided by Antonio Sala-Mirete).

## Results

### Phylogenetic analyses

The COI alignment comprised 43 sequences and was 658 bp long. The ITS2 alignment comprised 37 sequences and was 901 bp long. The final combined data set consisted of 1559 bp and 46 sequences.

**Figure 1.**
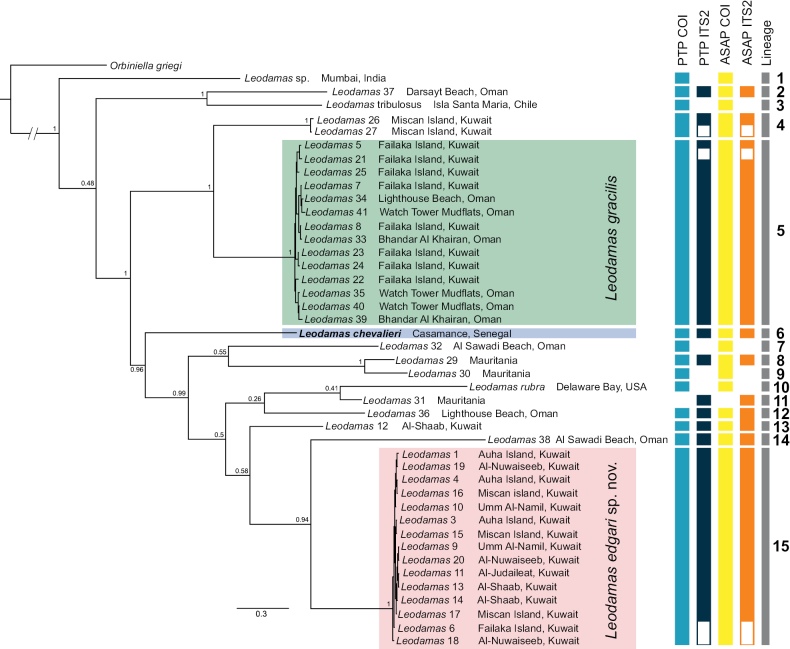
Bayesian inference (BI) based on the concatenated dataset of COI and ITS2. Bayesian posterior probabilities are shown on the nodes. Species delimitation results inferred by DNA-based methods are indicated by four colored bars to the right of the BI tree with the lineage number. White bars indicate missing data.

The topologies of the concatenated BI and ML trees were similar, both showing 15 lineages (Fig. 1, Suppl. material 2: fig. S1). The three COI sequences obtained from GenBank are confirmed as three different lineages, one of them corresponding to a potentially undescribed species from Mumbai, India (Lineage 1), and the other two belonging to two specimens identified as *L. tribulosus* (Lineage 3) and *L. rubra* (Lineage 10) in Bleidorn et al. (2009). Lineage 4 included two specimens from Miscan Island, Kuwait (PP = 1 and BS = 100%). Several specimens from different localities in Kuwait and Oman comprised the Lineage 5 (PP = 1 and BS = 100%), and several specimens from different localities in Kuwait, the Lineage 15 (PP = 1 and BS = 100%). Lineage 6 was represented by a specimen of *L. chevalieri* from Senegal (the type locality). The remaining lineages corresponded to eight singletons of potentially undescribed species from different localities of Kuwait (Lineage 13), Mauritania (Lineages 8, 9, and 11), and Oman (Lineages 2, 7, 12, and 14). The only differences between the concatenated BI and ML trees were the relationships among Lineages 10–13. In the BI, Lineages 10–12 formed a poorly supported group (PP = 0.26) and Lineage 13 was sister to Lineages 14 and 15 with low support (PP = 0.58). In the ML, Lineages 10 and 12 formed a poorly supported group (BS = 46%) and Lineages 11 and 13 another poorly supported group (BS = 55%). The topologies of the BI and ML trees of COI were nearly identical (Suppl. material 2: figs S2, S3), with the only difference in the position of Lineages 1, 2, and 3. In the COIBI, the group combining Lineages 2 and 3 was sister to the rest of lineages with a high support (PP =1), whilst in the COIML, Lineage 1 was sister to the rest of lineages with a high support (BS = 100%). Similarly, the topologies of the BI and ML trees of ITS2 were nearly identical (Suppl. material 2: figs S4, S5), with the only difference in the position of Lineage 8. In the ITS2 BI, Lineage 8 was sister to Lineage 15 with low support (PP = 0.40), whilst in the ITS2 ML, was sister to the group combining Lineages 11–14 (BS = 75%).

### Species delimitation and genetic distances

The PTP analysis for COI identified 14 putative species (here referred to as lineages), with species delimitation support > 0.990, corresponding to eleven of the twelve Arabian and southwestern African lineages, Lineage 1 (India), Lineage 3 (Chile), and Lineage 11 (USA) (Suppl. material 2: file S1: A). Lineage 12 was missing in the COI dataset. The PTP analysis for ITS2 recognized ten putative species, with support > 0.991, corresponding to ten of the 12 Arabian and southwestern African lineages (Suppl. material 2: file S1: B). Lineages 1, 3, 7, 9, and 11 were missing in the ITS2 dataset.

The best ASAP partition (i.e., showing the lowest score) for COI returned 13 putative species, corresponding to ten of the 12 Arabian and southwestern African lineages, and Lineages 1, 3, and 11 (Suppl. material 2: file S2: A). Lineages 8 and 9, both from Mauritania, recognized by PTP analysis as separate species, were merged into a single group. The best ASAP partition for ITS2 included 11 putative species, corresponding to ten of the 12 Arabian and southwestern African lineages (Suppl. material 2: file S2: B). Lineages 10 and 13 recognized by PTP analysis as separate species were merged into a single group, and Lineages 1, 3, 7, 9, and 11 were missing in the ITS2 dataset.

The uncorrected p-distances within and between lineages are summarized in Suppl. material 1: table SS2. The within lineage average p-distances varied from 0.08–0.95% in COI and from 0.10–0.36% in ITS2. The between lineage average p-distances were 8.21–25.70% in COI, and 4.82–24.34% in ITS2. Lineages 5 and 15 presented distances higher than 14% in COI and 6% in ITS2. The uncorrected p-distances based on 16S between Lineage 15 and *Leodamas australiensis* from Australia and from the Mediterranean Sea were 10.2% and 12.93%, respectively.

Combining altogether the results of the phylogenetic analyses, species delimitation, and genetic distances (Fig. 1, Suppl. material 2: figs S1–S5), we consider there to be eight distinct species (i.e., Lineages 2, 4, 5, 7, 10, 13, 14, and 15) in the Arabian region and four (i.e., Lineages 6, 8, 9, and 12) in western Africa.

### Systematic account

The integrative analysis of the Arabian and West African *Leodamas* material resulted in the assigning of the species name *Leodamas gracilis* to Lineage 5 and *Leodamas chevalieri* to Lineage 6. Below we provide detailed morphological description of the Arabian population of *L. gracilis* and a redescription of *L. chevalieri* based on the type material and non-type specimens collected close to the type locality. Lineage 15 is here described as a new species, *Leodamas edgari* sp. nov. For the other lineages, the specimens were too few or in poor condition to be described or named.

#### Orbiniidae Hartman, 1942

##### Leodamas

Taxon classificationAnimaliaAnnelidaOrbiniidae

Kinberg, 1866

19855B8B-12A3-5095-A2CC-D275AA56247D

Leodamas Kinberg, 1866: 252; Blake 2000: 448; Zhadan et al. 2015: 786 –789; Blake 2017: 48 –52 (in part).
Scoloplos (Leodamas) : Hartman 1957: 284 –285; Pettibone 1957: 160; Day 1973: 84; Eibye-Jacobsen 2002: 87.

###### Type species.

*Leodamas verax* Kinberg, 1886, by monotypy.

###### Diagnosis.

(emended from Zhadan 2020) Prostomium acutely pointed, usually prolonged. Peristomium with one achaetous peristomial ring. Branchiae simple or branched, usually present from chaetiger 5–7 and rarely from chaetiger 12. Posterior thoracic neuropodia may bear one or two podal papillae and one or two subpodial papillae. Abdominal neuropodia bilobed with reduced inner lobe. Thoracic neuropodia bearing large and numerous uncini accompanied by few crenulated capillaries, crenulated capillaries sometimes absent. Abdominal neuropodia possess robust protruding aciculae. Abdominal forked notochaetae present or absent.

###### Remarks.

Zhadan et al. (2015) and Zhadan (2020) suggested the presence of protruding robust aciculae in abdominal neuropodia as the main diagnostic character of *Leodamas*, whilst Blake (2017, 2020, 2021) proposed the presence of large and conspicuous uncini in the thoracic neuropodia as a diagnostic character and transferred several species of *Scoloplos* with such uncini to *Leodamas*. A phylogenetic study of Orbiniidae based on genome skimming (Meca 2025) showed the presence of large and conspicuous uncini in thoracic neuropodia being a homoplastic character found in species from four unrelated clades. In the same study, a single protruding aciculum in the abdominal neuropodia was only found in the species belonging to *Leodamas* sensu lato, whilst one to three embedded aciculae in the abdominal neuropodia were only found in the species from *Scoloplos* sensu lato. Thus, we follow Zhadan’s (2020) diagnosis which is here emended, with the addition of the character “shape of abdominal neuropodia” since all *Leodamas* species exhibit bilobed abdominal neuropodia with a reduced inner lobe which is replaced by the protruding robust aciculae.

The species of *Scoloplos* transferred by Blake (2017) to *Leodamas*, as well as the recently described *Leodamas perissobranchiatus* Blake, 2017 and *Leodamas bathyalis* Blake, 2020, showing a single embedded aciculum in abdominal neuropodia are here considered as belonging to *Scoloplos* until their phylogenetic position within Orbiniidae is assessed based on molecular data.

##### Leodamas chevalieri

Taxon classificationAnimaliaAnnelidaOrbiniidae

(Fauvel, 1902)

5AD52DCA-3E58-563C-BAAD-1D21F3756CF2

Figs 2, 3, 4, 5, 6

Aricia chevalieri Fauvel, 1902: 83–86, figs 23–28.
Non Aricia chevalieri: Gravier 1906: 167, pl. II, figs 193–195; Fauvel 1919; 428–429.
Non Scoloplos (Leodamas) chevalieri: Wesenberg-Lund 1949: 322; Fauvel 1953: 308–309, fig. 161; Amoureux 1976: 23; Ong 1995: 271, 273, 274, fig. 9.
Non Leodamas chevalieri: Al-Kandari et al. 2019: 7.

###### Type material.

***Lectotype*** here designated • MNHN-IA-2000-2122. ***Paralectotypes***: • MNHN-IA-2000-2123 (two incomplete paralectotypes); MNHN-IA-2001-160 (numerous abdominal fragments).

###### Other material examined.

• ZMBN 115302 (1 spm); ZMBN 115304 (3 juveniles); ZMBN 159076 (1 spm, DNA voucher Leo43); ZMBN 159077 (1 spm); ZMBN 159078 (1 spm); ZMBN 159079 (1 spm).

###### Diagnosis.

One podal papilla in posteriormost thoracic chaetigers; thoracic neuropodia with five rows of uncini accompanied by few capillary chaetae; abdominal neuropodia with single, slightly bent, projecting aciculum.

###### Type locality.

**Figure 2.**
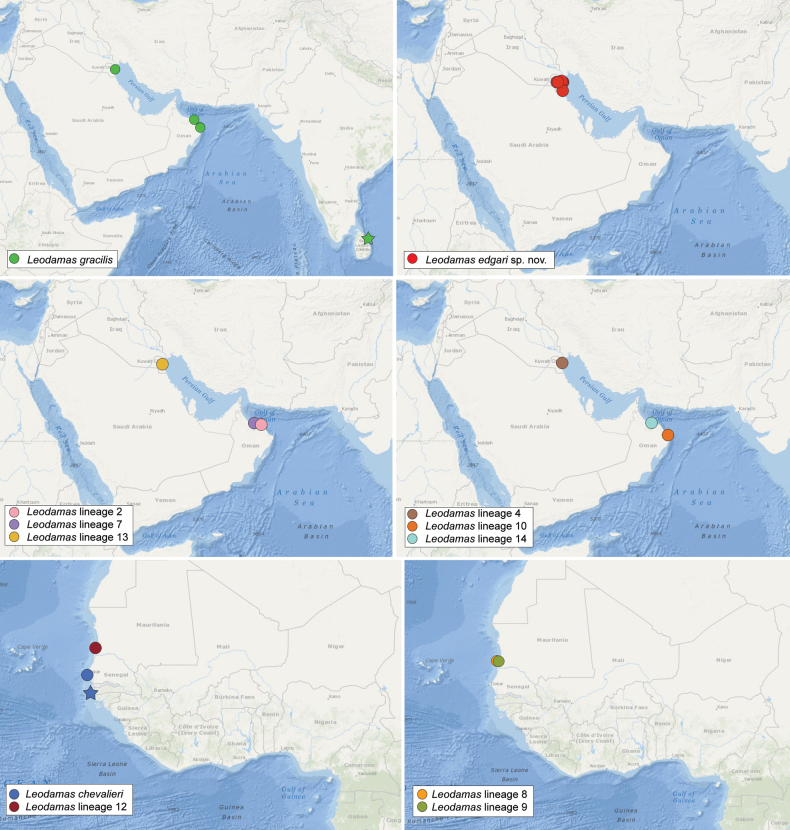
Distributional maps of *Leodamas* species. Stars represent the type localities of the species.

Estuary of Casamance River, Senegal, 6 km from the mouth, 12.589776, -16.703020, in intertidal fine sand, slightly muddy, around rotten wood and other debris (Fig. 2).

###### Description (based on the type material).

**Figure 3.**
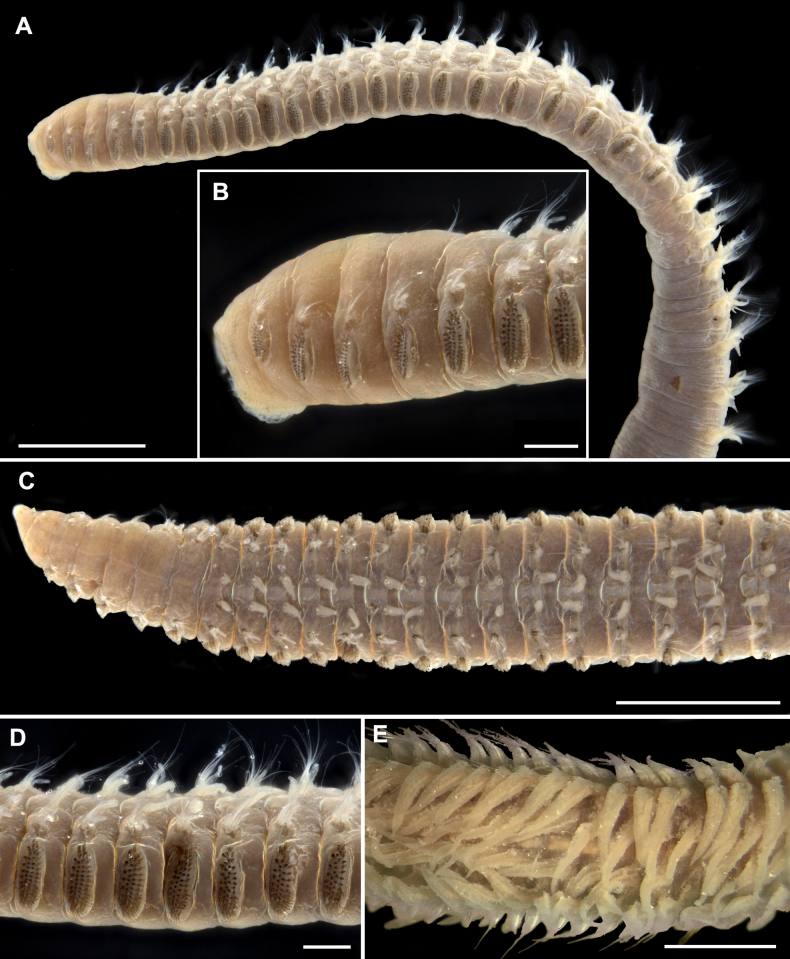
*Leodamas chevalieri*, type specimens, light microscopy, stereomicroscope; **A–D**. Lectotype, MNHN-IA-TYPE0728; **E**. Paralectotype. **A**. General view from left side; **B**. Anterior part from left side, prostomium is not seen; **C**. Thorax, dorsal view; **D**. Middle thorax, lateral view; **E**. Abdomen, dorsal view. Scale bars: 1 mm (**A, C**); 200 µm (**B, D**); 500 µm (**E**).

**Figure 4.**
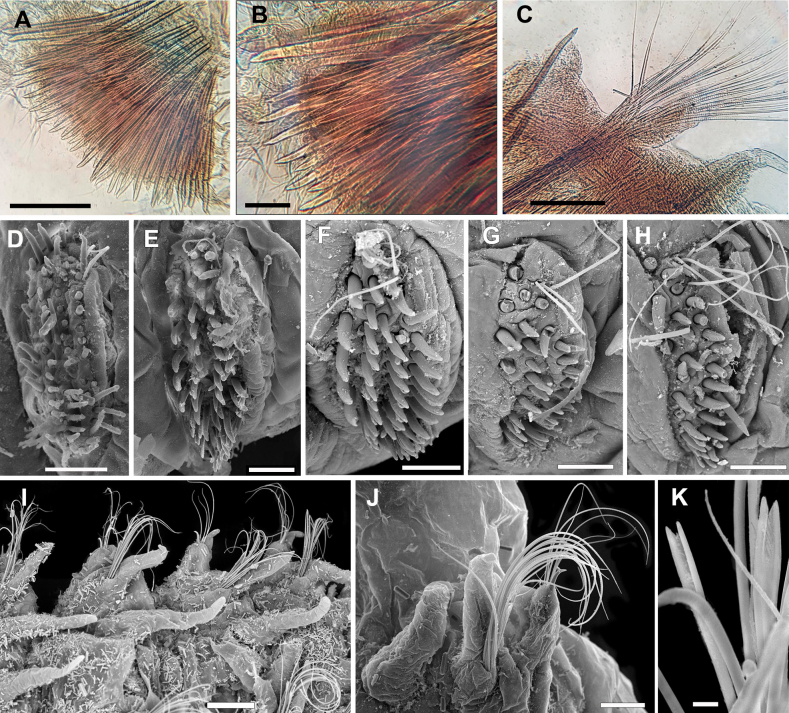
*Leodamas chevalieri*, paralectotypes. **A–C**. Light microscopy, compound microscope; **D–J**. SEM. **A, B**. Thoracic neurochaetae; **C**. Abdominal parapodium; **D**. Neuropodium of chaetiger 2; **E**. Neuropodium of chaetiger 12; **F**. Neuropodium of chaetiger 16; **G**. Neuropodium of chaetiger 20; **H**. Neuropodium of chaetiger 21; **I, J**. Abdominal parapodia; **K**. Forked chaetae from abdominal notopodium. Scale bars: 100 µm (**A, C**); 50 µm (**B, D–H, J**); 150 µm (**I**); 6 µm (**K**).

Lectotype incomplete with 73 chaetigers, 44 mm in length and 0.8 mm in width of thorax. Body long, slender; thorax flattened dorso-ventrally, inflated in anterior part; abdomen cylindrical (Fig. 3A). Color in ethanol whitish-yellow, chaetae brown. Prostomium conical, pointed (Fig. 3B). Nuchal organs not seen. Thoracic width up to 0.9 mm in paralectotypes. Thoracic chaetigers numbering 22 (22 and 23 in paralectotypes). Branchiae starting from chaetiger 6, triangular with broad bases and tapering tips; gradually increasing in size; in abdomen becoming long, digitate, equal in length or slightly shorter than notopodia (Fig. 3C, E). Thoracic postchaetal notopodial lobes developed from chaetiger 2, lanceolate, gradually increasing in size; in anterior abdomen lanceolate, shorter than branchiae; in posterior abdomen becoming very long, digitate, equal in length or slightly longer than branchiae (Figs 3D, 3E, 4C, 4H, 4I). Thoracic neuropodial postchaetal lobes as low ridges, in posteriormost one or two thoracic chaetigers with low triangular podal papilla (Figs 3D, 4G). Abdominal neuropodia rectangular, with long triangular outer lobe and reduced inner lobe with projecting aciculum (Fig. 4C, H, I). Parapodial flange not developed. Subpodal, stomach, and flange papillae absent. Dorsal organs not seen.

Notopodia bearing crenulated capillary chaetae in both thorax and abdomen; abdominal notopodia additionally having forked chaetae with subequal tines and pointed tips (Fig. 4K). Thoracic neuropodia with five rows of uncini, posterior neuropodia with three or four rows; posteriormost row short, developed only in ventral part of neuropodia, curved under antecedent row (Fig. 4D–G). In anterior and posterior thorax, anteriormost row of uncini developed only in dorsal part of neuropodia (Fig. 4F, G).

In anterior thorax, very thin and short single capillary chaeta located dorsally in forth row; in posterior thorax, up to three or four capillaries (Fig. 4F, G). Uncini in anterior row thicker than in posterior; hooded, slightly bent, with rounded conical tips; mostly smooth, but in chaetiger 2 serrated on anterior surface; in chaetiger 16 uncini bearing longitudinal groove subdistally (Fig. 4E). Abdominal neuropodia with bundle of capillary chaetae and projecting aciculum, its distal end slightly bent (Fig. 4C, H, I). Pygidium unknown.

###### Remarks on morphology of non-type material collected off Senegal.

The morphology of the non-type specimens agrees well with that of the type specimens, with slight differences in some characters. The main characteristics of the type and non-type material are summarized in Table 1.

**Table 1.** Comparison between the type and non-type material of *Leodamas chevalieri*. Abbreviations: CH, Chaetiger; TCH, Thoracic chaetiger.

	Type material	Non-type material (adult specimens)
Number of thoracic chaetigers	22–23	18–19
Thoracic width (mm)	0.7–0.9	0.85
Capillaries in thoracic neuropodia	In all TCH, 2 in anterior thorax, 1 in middle thorax, 3 or 4 in posterior thorax	In all TCH, up to 3 in anterior and posterior thorax, 1 in middle thorax
Thoracic uncini	Five rows, last row only ventral, curved. Posterior TCH with 3 rows only. First row of uncini in anterior and posterior TCH only dorsal	Five rows, last row only ventral, curved ventrally under fourth row. Posterior TCH with 3 rows only. First row of uncini in anterior and posterior TCH only dorsal but slightly longer than in type specimens
Uncini serration	Mostly smooth, serration seen only in CH 2 or 3 in 2–5 rows, with 8 or 9 ribs	Smooth in anterior row, with 2–7 ribs in 2–4 rows, with 8 or 9 in 5^th^ row. With clear serration in juveniles

**Figure 5.**
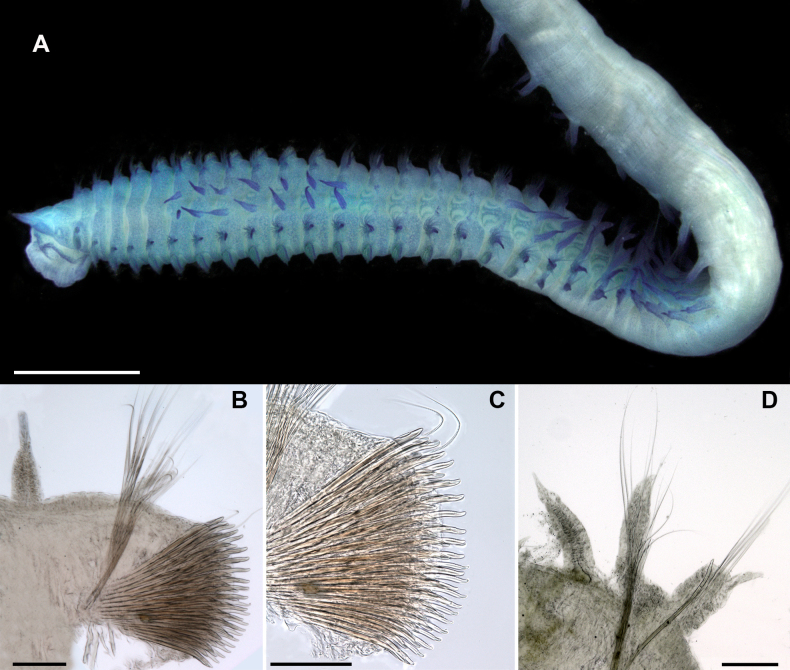
*Leodamas chevalieri*, non-type specimen, ZMBN 115302, Senegal, light microscopy. **A**. General view; **B**. Parapodium of chaetiger 8; **C**. Same, neuropodium; **D**. Abdominal parapodium. Scale bars: 1 mm (**A**); 100 µm (**B–D**).

**Figure 6.**
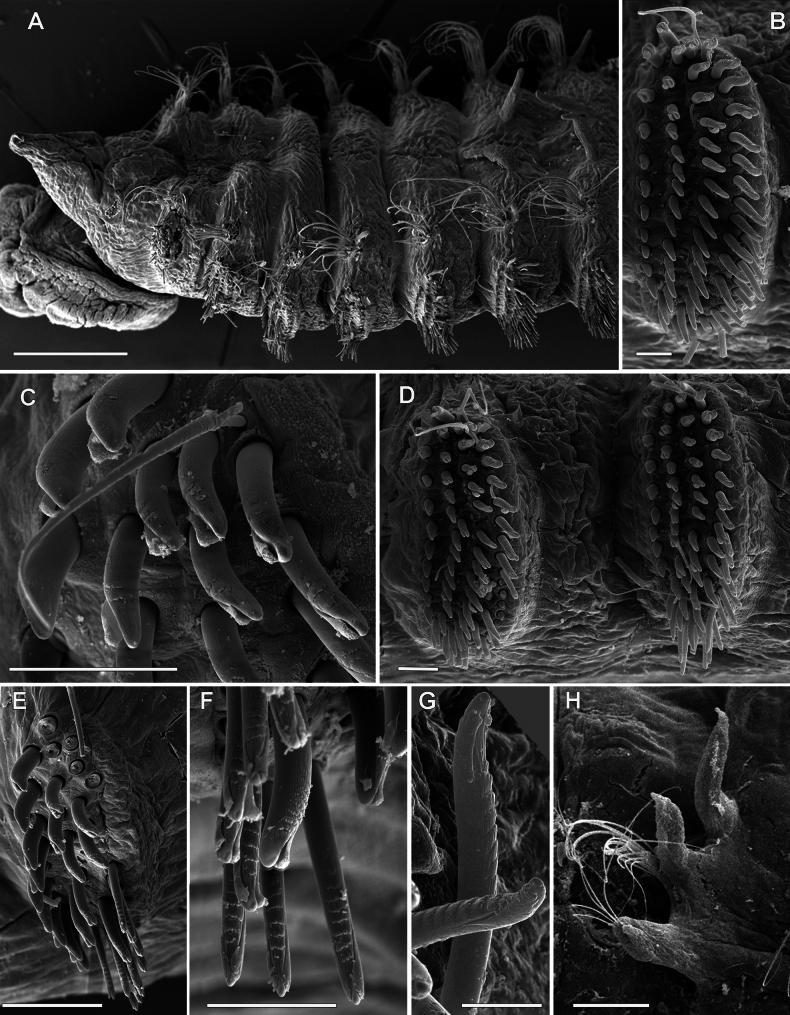
*Leodamas chevalieri*, non-type specimen, ZMBN 115302, Senegal, SEM. **A**. Anterior end, dorso-lateral view; **B**. Neuropodium of chaetiger 7; **C**. Same, upper uncini; **D**. Neuropodia of chaetigers 8 and 9; **E**. Neuropodium of chaetiger 16; **F**. Same, lower uncini; **G**. Same lower uncinus; **H**. Abdominal neuropodia. Scale bars: 250 µm (**A**); 20 µm (**B, F**); 10 µm (**G**); 30 µm (**C, D**); 50 µm (**E**); 100 µm (**H**).

The number of thoracic chaetigers is lower than in the type material, i.e., 18 (10–12 in juveniles) (Fig. 5A). Branchiae are longer than notopodia in the anterior abdomen and equal in length or slightly shorter in the middle abdomen (Figs 5A, 5B, 5D, 6H), instead of equal in length or slightly shorter than notopodia throughout abdomen as in the type specimens. Thoracic postchaetal notopodial lobes are digitate and abdominal lobes are lanceolate (Figs 5B, 5D, 6H), instead of lanceolate thoracic notopodial lobes and digitate abdominal lobes observed in the type specimens.

Dorsal organs were observed only in the non-type specimens, starting from chaetiger 8 as two horseshoe-shaped ciliated stripes (Fig. 5A).

The anteriormost row of uncini is developed only in dorsal part of neuropodia in anterior and posterior thorax as in the type specimens but is slightly longer (Fig. 6A, B, E). One to three capillary chaetae located dorsally in all thoracic neuropodia (Figs 5C, 6B, 6D, 6E), whilst up to four in the type specimens in posterior thorax. All uncini, except the anteriormost row, are serrated, with 2–7 low transverse crenulated ribs in the middle rows, and eight or nine ribs in the posteriormost row (Figs 5C, 6C, 6F, 6G). In the type specimens, most of uncini are smooth; only in chaetigers 2 and 3 uncini from the 2^nd^ to the 5^th^ row have eight or nine ribs. Moreover, uncini of the type specimens in chaetiger 16 show a longitudinal groove subdistally, which was not observed in the non-type material.

###### Remarks.

Since the original description by Fauvel (1902) based on material collected from the estuary of the Casamance River (Senegal, West Africa), other populations of *Leodamas chevalieri* have been described from different areas, with some morphological differences discussed below. *Leodamas chevalieri* is similar to several species inhabiting the East Atlantic, including the Mediterranean Sea, and the Indo-West Pacific, including the Arabian region (i.e., *L. australiensis*, *L. dubius* (Tebble, 1955), and *Leodamas edgari* sp. nov.) in having one podal papilla in the posteriormost thoracic neuropodia, five rows of uncini accompanied by few capillaries in thoracic neuropodia, and a single, curved projecting aciculum in abdominal neuropodia.

However, *L. chevalieri* differs from *L. australiensis*, *L. dubius*, and *L. edgari* sp. nov. in the degree of curvature of the aciculum in abdominal neuropodia, being slightly curved in *L. chevalieri*, and strongly hooked in some segments of *L. australiensis*, *L. dubius*, and *L. edgari* sp. nov.

There were two main differences in the morphology of *L. chevalieri* from various localities – the presence of forked chaetae in abdominal notopodia and smooth versus serrated thoracic neuropodial uncini. In the original description (Fauvel 1902), forked chaetae were not mentioned, and the thoracic neuropodial uncini were described as smooth. Wesenberg-Lund (1949) also did not report forked chaetae in the specimens from the Arabian Gulf. Harmelin (1969: Plate 1, Fig. 10), in the description of *L. chevalieri candiensis* from the Mediterranean Sea, also noted the absence of forked chaetae in the holotype, but they were present in the paratypes and are illustrated in his figure. He reported the uncini in *L. chevalieri candiensis* being serrated. Gravier (1906) described *L. chevalieri* from the Red Sea and reported 19–26 thoracic chaetigers, with forked chaetae present, and uncini having seven or eight ribs. Blake (2017: 65, Table 1) considered the absence of forked chaetae as a unique character for *L. chevalieri*. He also described the uncini as being straight, narrowing at the tip, blunt, with a lateral hood.

Our examination of both type and non-type specimens from Senegal confirmed the presence of forked chaetae in abdominal notopodia. They have the typical appearance of *Leodamas*, with subequal tines and pointed tips. The situation with serration of uncini was more complex. In the type specimen studied under SEM, the uncini were mostly smooth, serration was seen only in thoracic chaetigers 2 and 3 in 2–5 rows where the uncini bore eight or nine ribs. In the non-type specimens from Senegal, serration was much more prominent, present in all thoracic chaetigers, in 2–5 rows, from 2–7 to 8 or 9 ribs. Also, the differences in thickness between uncini in the first row and in more posterior rows appeared to be more pronounced in the non-type specimens. This different degree of serration in uncini between type and non-type specimens could be seen as an intraspecific variation.

###### Distribution.

Coastal areas of Senegal, from intertidal to 30 m (Fig. 2).

##### Leodamas edgari sp. nov.

Taxon classificationAnimaliaAnnelidaOrbiniidae

3F464663-B941-50C6-815B-68E976461DF2

https://zoobank.org/FFD766FA-FBAA-46F7-B80E-A7377D3449ED

Figs 2, 7–11

###### Type material examined.

***Holotype***: • ZMMU WS14151 (DNA voucher Leo59). ***Paratypes***: • ZMMU WS8279 (1 paratype); WS8281 (1 paratype); ZMMU WS8283 (6 paratypes); WS8284 (2 paratypes); WS8291 (12 paratypes); ZMMU WS8299 (1 paratype); P12378 (1 paratype); ZMMU WS14106 (64 paratypes); ZMMU WS14157 (4 paratypes); ZMMU WS14164 (6 paratypes); ZMMU WS20583 (1 paratype, DNA voucher Leo15E); ZMMU WS20584 (1 paratype, DNA voucher Leo17E); ZMMU WS20585 (1 paratype, DNA voucher Leo18E); ZMMU WS20587 (1 paratype, DNA voucher Leo20E); ZMMU WS20590 (1 paratype, DNA voucher Leo57); ZMMU WS20591 (1 paratype, DNA voucher Leo58); ZMMU WS20593 (1 paratype, DNA voucher Leo61); ZMMU WS20594 (1 paratype, DNA voucher Leo62); ZMMU WS20595 (1 paratype, DNA voucher Leo63); ZMMU WS20596 (1 paratype, DNA voucher Leo64); ZMMU WS20597 (1 paratype, DNA voucher Leo65); ZMMU WS20598 (1 paratype, DNA voucher Leo32E); ZMMU WS20600 (1 paratype, DNA voucher Leo34E).

###### Other material examined.

• ZMMU WS20599 (E-voucher LEODA025-26); ZMMU WS8300 (6 spms).

###### Diagnosis.

One podal papilla in posteriormost thoracic chaetigers; thoracic neuropodia with five rows of uncini accompanied by few capillaries; abdominal neuropodia with single, curved or strongly hooked projecting aciculum.

###### Type locality.

Al-Judailiat, Kuwait, 29.379322, 47.764992, intertidal, muddy sand.

###### Description (based on the type material).

**Figure 7.**
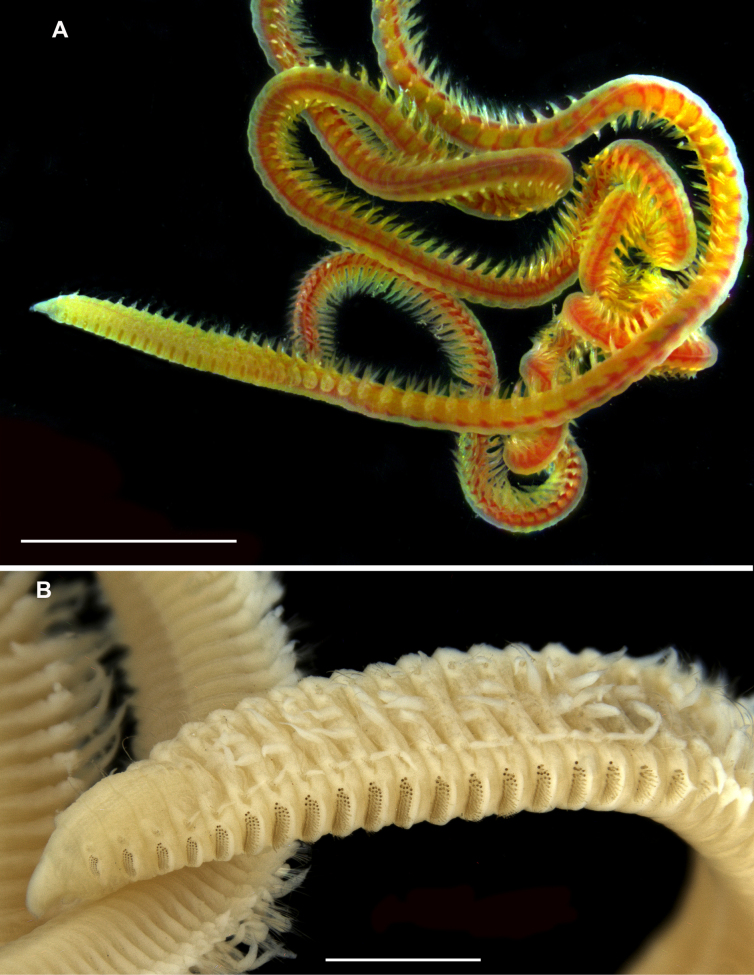
*Leodamas edgari* sp. nov., holotype, ZMMU WS14151, light microscopy. **A**. Living specimen; **B**. Anterior end, dorso-lateral view. Scale bars: 5 mm (**A**); 1 mm (**B**).

**Figure 8.**
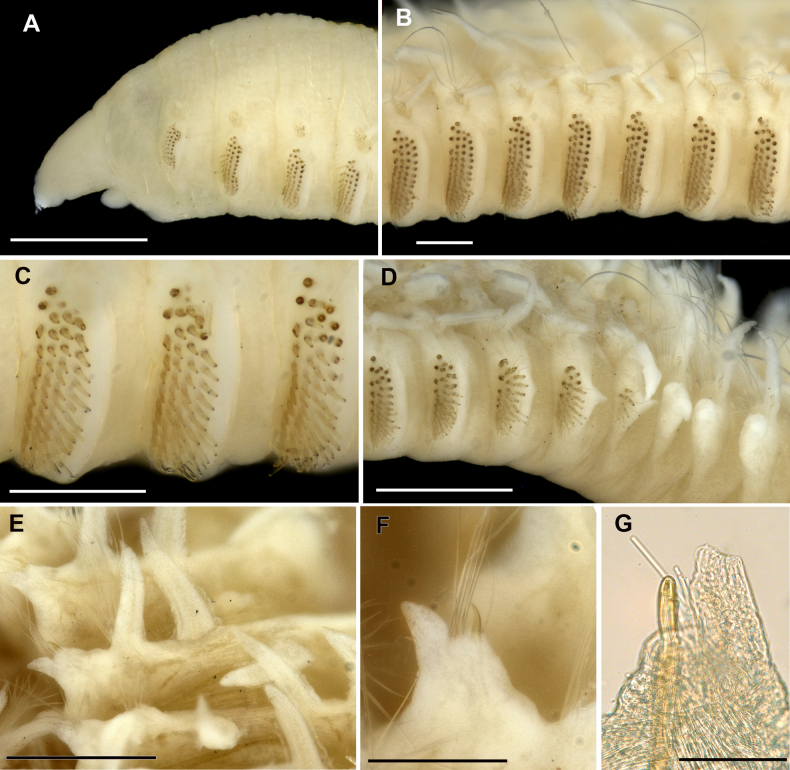
*Leodamas edgari* sp. nov., holotype, ZMMU WS14151, light microscopy. **A**. Anterior end, lateral view; **B**. Chaetigers 10–16, dorso-lateral view; **C**. Neuropodia of chaetigers 10–12, lateral view; **D**. Thorax-abdomen transition, dorso-lateral view; **E**. Abdominal parapodia, chaetigers 82–84; **F**. Neuropodia of chaetiger 82; **G**. Neuropodia of chaetiger 87. Scale bars: 500 µm (**A, D, E**); 200 µm (**B, C, F**); 100 µm (**G**).

**Figure 9.**
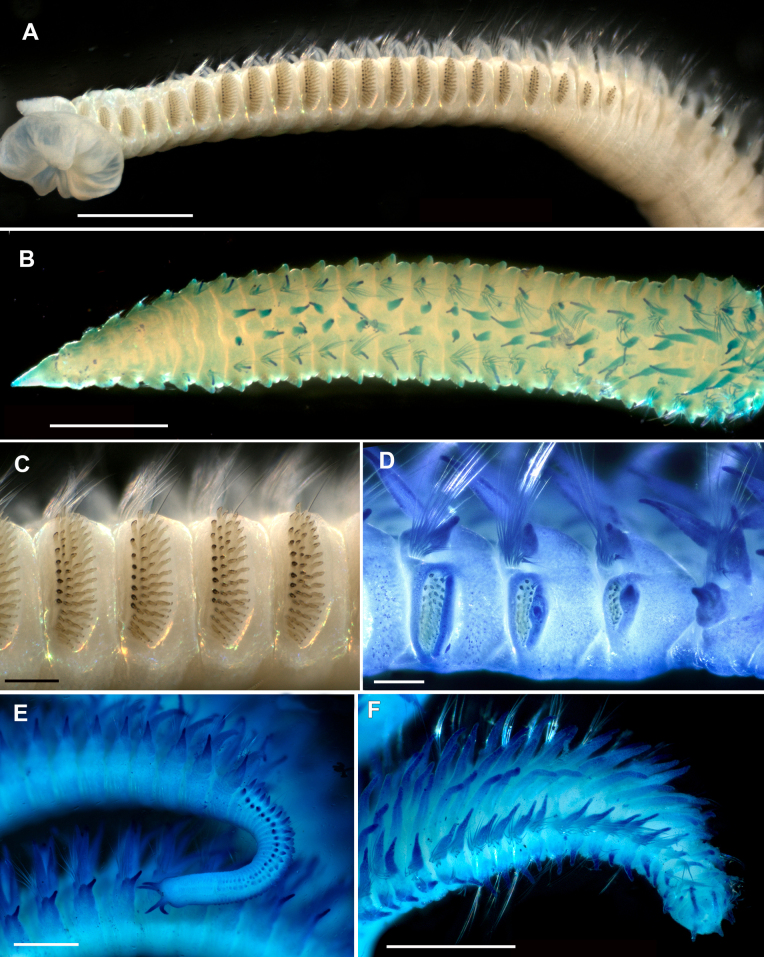
*Leodamas edgari* sp. nov., paratypes, light microscopy. **A**. ZMMU WS8279, anterior end, lateral view; **B**. P12378, dorsal view; **C**. Same as **A**., neuropodia of chaetigers 10–12, lateral view; **D**. ZMMU WS8300, thorax-abdomen transition, lateral view; **E**. ZMMU WS8281, posterior end with regenerating pygidium; **F**. ZMMU WS8283, posterior end with pygidium. Scale bars: 1 mm (**A, B**); 200 µm (**C, D**); 500 µm (**E, F**).

Holotype complete with 336 chaetigers (128–257 in paratypes), 55 mm in length (16–42 mm in paratypes) and 1.3 mm of thoracic width (0.9–1.4 mm in paratypes). Body long, slender; thorax flattened dorso-ventrally, inflated in anterior part; abdomen cylindrical (Figs 7A, 9A, 11A). Color in ethanol whitish-yellow, chaetae brown; live worms yellow with red blood vessels seen through body wall. Thoracic chaetigers numbering 20 (16–21 in paratypes). Prostomium conical, pointed, peristomium as long as chaetiger 1 (Figs 7B, 8A, 9A, 9B, 11B). Nuchal organs on anterior border of peristomium as transverse ciliated slits (Fig. 11B).

**Figure 10.**
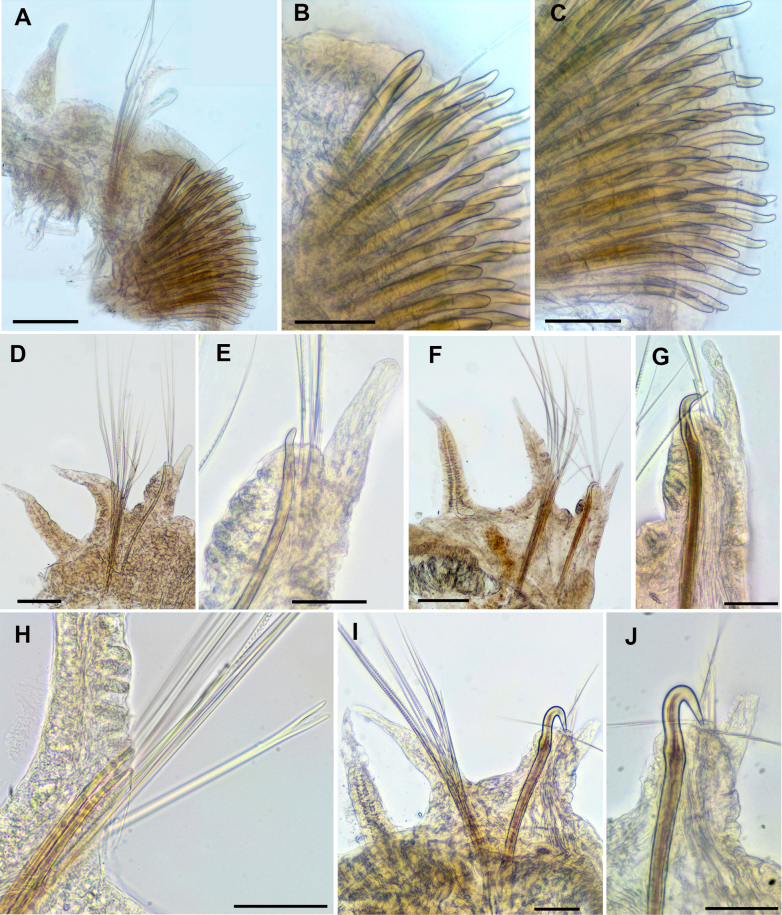
*Leodamas edgari* sp. nov., paratypes, ZMMU WS8283, parapodia slides. **A**. Parapodium of chaetiger 7; **B**. Same, upper neurochaetae; **C**. Same, lower neurochaetae; **D**. Parapodium of chaetiger 24 (anterior abdomen); **E**. Same, neuropodium; **F**. Posterior abdominal parapodium with slightly curved aciculum; **G**. Same, neuropodium; **H**. Posterior abdominal notopodium, two aciculae and forked chaetae seen; **I**. Posterior abdominal parapodium with strongly hooked aciculum; **J**. Same, neuropodium. Scale bars: 100 µm (**A, D, F, I**); 50 µm (**B, C, E, G, H, J**).

**Figure 11.**
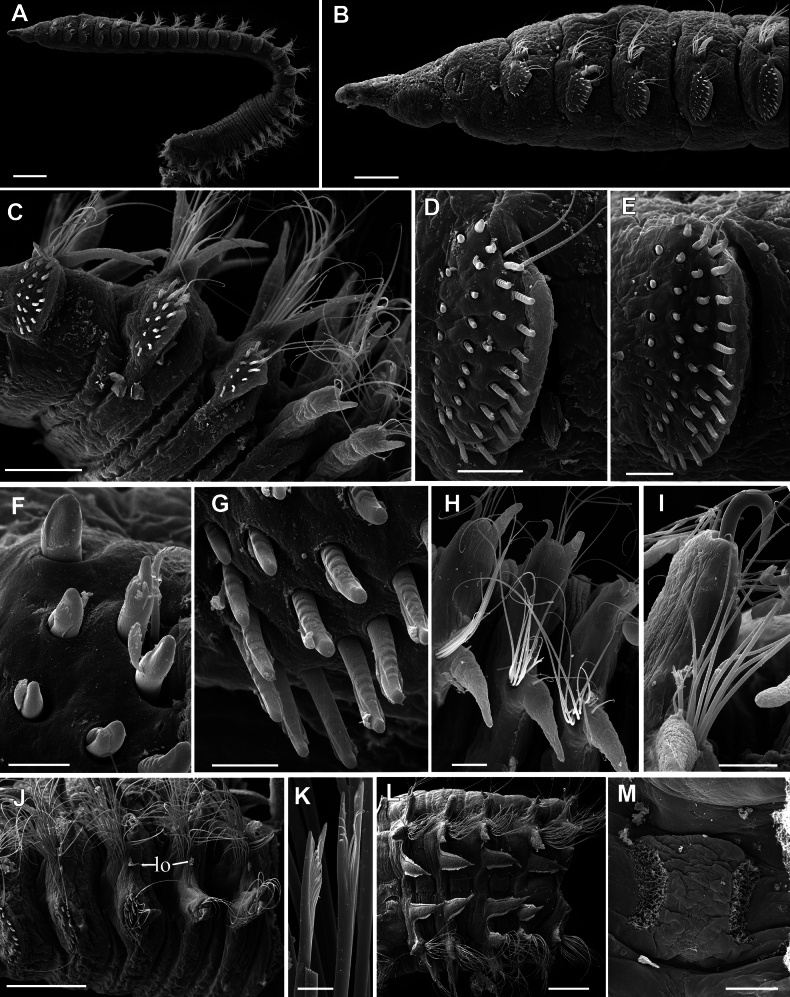
*Leodamas edgari* sp. nov., paratypes, SEM. **A–I**. ZMMU WS8283, **J–M**. ZMMU WS8279. **A**. General lateral view; **B**. Anterior end, lateral view; **C**. Thorax-abdomen transition, chaetigers 15–19; **D**. Neuropodium of chaetiger 4; **E**. Neuropodium of chaetiger 9; **F**. Upper uncini of chaetiger 11; **G**. Lower uncini of chaetiger 11; **H**. Abdominal parapodia; **I**. Abdominal notopodium and neuropodium with strongly hooked aciculum; **J**. Chaetigers 19–23, thorax-abdomen transition, lateral organs (lo) are seen; **K**. Forked chaetae; **L**. Abdominal segments, dorsal view; **M**. Dorsal organ. Scale bars: 300 µm (**A**); 100 µm (**B, C**); 30 µm (**D, E, H, I**); 10 µm (**F, G**); 250 µm (**J, L**); 10 µm (**K**); 50 µm (**M**).

Branchiae from chaetiger 6, triangular with drawn out tips; gradually increasing in size; in abdomen becoming long, digitate, longer than notopodia; ciliated on both sides excluding tips (Figs 7B, 8E, 9B, 10D, 10F, 11L).

Thoracic notopodial postchaetal lobes developed from chaetiger 2 (2 or 3 in paratypes), as small papillae; gradually increasing in size; in anterior abdomen lanceolate, shorter than branchiae; in posterior abdomen becoming long, digitate, shorter than branchiae; ciliated on inner side (Figs 8E, 9D, 10A, 10D, 10F, 10I, 11C). Branchial bases connected with notopodial bases by low ridge with weak ciliation.

Thoracic neuropodial postchaetal lobes as low ridges, in posterior thorax, one or two posteriormost chaetigers with low triangular podal papilla (Figs 8B, 8C, 8D, 9C, 9D, 11C, 11D, 11E, 11J). Abdominal neuropodial lobes rectangular, with long cirriform-triangular outer lobe and reduced inner lobe with single projecting curved or hooked aciculum (Figs 8F, 8G, 10D, 10E, 10F, G I, 10J, 11H, 11I). Parapodial flange not developed. Subpodal, stomach, and flange papillae absent. Ciliated lateral organs located between notopodial and neuropodial bases (Fig. 11J).

Dorsal organs as curved oval ciliated pits located mid-dorsally (Fig. 11M).

Notopodia bearing crenulated capillary chaetae in both thorax and abdomen; abdominal notopodia with two thin aciculae; additionally with forked chaetae with unequal tines, pointed tips and crenulated handles similar to that in capillary chaetae (Figs 10H, 11K).

Thoracic neuropodia with five rows of uncini and 1–3 thin capillaries disposed dorsally; first row of uncini shifted dorsally, slightly bent over second row; fifth row of uncini developed only in ventral part, bent under preceding rows (Figs 8B, 8C, 8D, 9C, 11D, 11E). Uncini with rounded or pointed conical tips, slightly bent, some uncini with membranous hood, other non-hooded (Figs 10B, 10C, 11F). Uncini in anterior row smooth, thicker, and shorter than in posterior row where slightly or clearly serrated with up to 7 ribs (Fig. 11D, E, F, G). Worms of smaller size with more serrated uncini than larger specimens. Abdominal neuropodia with bundle of capillary chaetae and single projecting aciculum with distal end curved or strongly hooked. Degree of curvature of neuropodial acicula differs between segments and varies from slightly curved to bent 180° in holotype and paratypes (Figs 8F, 8G, 10D–G, 10I, 10J, 11H, 11I).

Pygidium with four short anal cirri, two dorsal and two ventral, anus terminal (Fig. 9E, F).

###### Remarks.

*Leodamas edgari* sp. nov. is similar to a group of species: *L. australiensis*, *L. chevalieri*, and *L. dubius* in having one podal papilla in posteriormost thoracic chaetigers, five rows of uncini accompanied by few capillaries in thoracic neuropodia and a single, curved, projecting aciculum in the abdominal neuropodia. While *L. edgari* sp. nov. is easily distinguishable from *L. chevalieri* by the presence of strongly hooked aciculae in some abdominal neuropodia, the differences between *L. edgari* sp. nov., *L. australiensis* and *L. dubius* are subtle. The main difference between *L. edgari* sp. nov. and *L. dubius* is the branchiae starting at chaetiger 6 in the former instead of chaetiger 7 in the latter. *Leodamas edgari* sp. nov. also shows less capillaries in the thoracic neuropodia (1–3 dorsal capillaries) than in *L. dubius* (4–8 capillaries forming half a row in the dorsal part of a neuropodium). Of all the species mentioned above, *L. edgari* sp. nov. and *L. australiensis* are the closest morphologically, with only subtle differences visible under SEM. In *L. edgari* sp. nov., the forked chaetae have clearly unequal tapering tines, whilst in *L. australiensis*, the tines are subequal. *Leodamas edgari* sp. nov. bears a membranous hood in some thoracic uncini, whilst *L. australiensis*, possesses very thick hoods on neuropodial uncini of posterior thorax. *Leodamas australiensis* was originally described from the western Australia (Hartmann-Schröder 1979) and then was subsequently reported from the Mediterranean Sea (Sala-Mirete et al. 2023). Apart from the subtle morphological differences, genetic distances in 16S between *L. edgari* sp. nov. and *L. australiensis* from Australia and from the Mediterranean indicate them being separate species (p-distance 10.2–12.93%).

###### Distribution.

Kuwait Bay, Auha, Failaka, and Miscan islands (Fig. 2).

###### Habitat.

Intertidal and upper subtidal zones, on muddy and sandy sediments.

###### Etymology.

The species name is dedicated to Edgar Meca Jimenez (the first author’s brother), in recognition of his courage and determination through personal adversities.

##### Leodamas gracilis

Taxon classificationAnimaliaAnnelidaOrbiniidae

(Pillai, 1961)

BE5CDFC6-D527-5717-83CF-99E0F889DC82

Figs 2, 12, 13

Scoloplos (Leodamas) gracilis Pillai, 1961: 22–24, figs 7M–O, 8A–F; Gallardo 1968: 94, pl. XL, figs 4–11; Eibye-Jacobsen 2002: 91–93, fig. 9A–D.

###### Type material examined.

• NHM 1960.3.13.12 (1 paratype).

###### Other material examined.

• ZMMU WS8293 (5 spms); ZMMU WS8292 (1 spm); ZMMU WS14160 (1 spm); ZMMU WS14162 (46 spms); ZMMU WS14686 (11 spms); ZMMU WS20586 (1 spm, DNA voucher Leo19); ZMMU WS20588 (1 spm, DNA voucher Leo55); ZMMU WS20589 (1 spm, DNA voucher Leo56); ZMMU WS20601 (1 spm, DNA voucher Leo35E); ZMMU WS20602 (1 spm, DNA voucher Leo36E); ZMMU WS20603 (1 spm, DNA voucher Leo37E); ZMMU WS20604 (1 spm; DNA voucher Leo67); ZMMU WS20605 (1 spm, DNA voucher Leo39E); ZMMU WS25019 (1 spm on SEM stub); P12379 (1 spm); Annelida 012042 (1 spm, DNA voucher Leo22); Annelida 012052 (1 spm, DNA voucher Leo28); Annelida 012055 (1 spm, DNA voucher Leo23); Annelida 012059 (1 spm, DNA voucher Leo24); Annelida 012060 (1 spm, DNA voucher Leo30); Annelida 012061 (1 spm, DNA voucher Leo29).

###### Diagnosis.

One podal papilla in posteriormost thoracic chaetigers; thoracic neuropodia with four rows of uncini, with anteriormost row up to two or three times thicker than the other three, accompanied by few capillary chaetae; abdominal neuropodia with single, straight, projecting aciculum.

###### Type locality.

Tambalagam Bay, Sri Lanka, 8.519585, 81.152620, at the center of the bay (Fig. 2).

###### Description.

**Figure 12.**
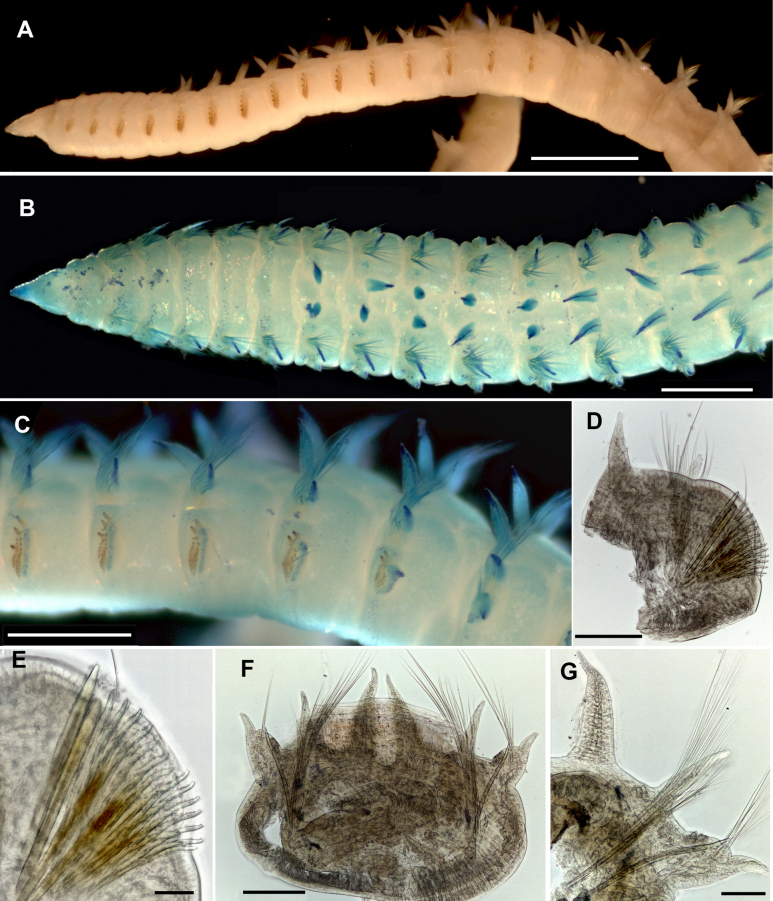
*Leodamas gracilis*, P12379, light microscopy. **A–C**. Stereomicroscope; **D–G**. Compound microscope. **A**. General lateral view; **B**. Anterior end, dorsal view; **C**. Thorax-abdomen transition, lateral view; **D**. Thoracic parapodia; **E**. Same, neurochaetae; **F**. Abdominal segment; **G**. Abdominal parapodia. Scale bars: 1 mm (**A**); 500 µm (**B, C**); 200 µm (**D, F**); 50 µm (**E**); 100 µm (**G**).

**Figure 13.**
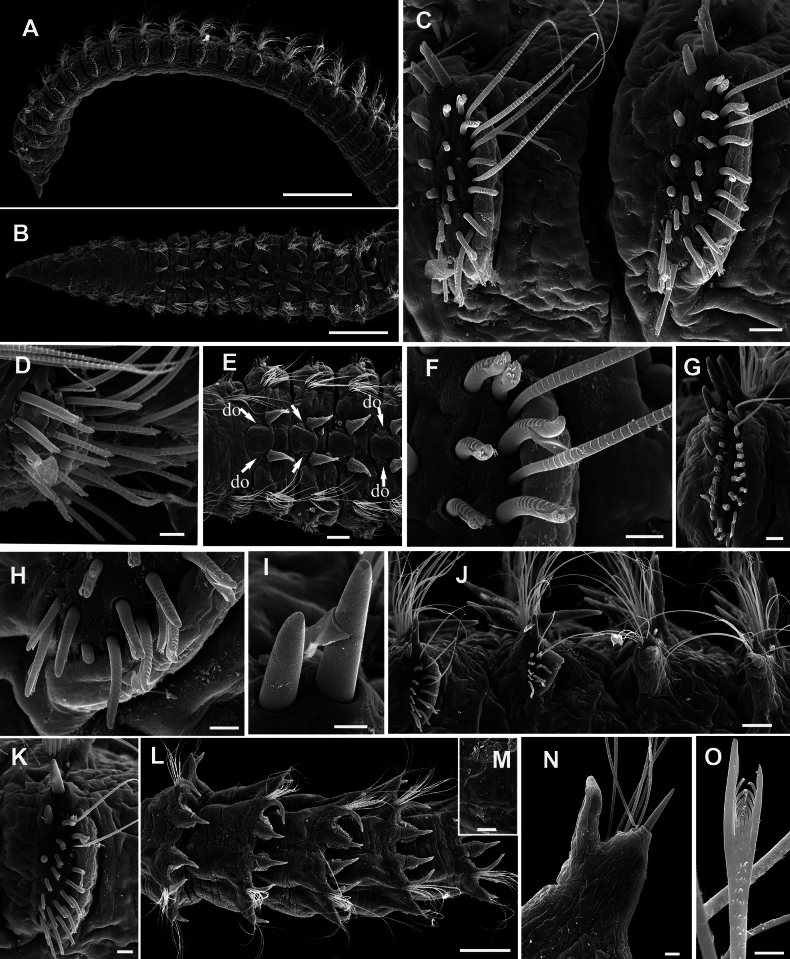
*Leodamas gracilis*, ZMMU WS8293, SEM. **A**. Anterior end, lateral view; **B**. Anterior end, dorsal view; **C**. Neuropodia of chaetigers 4 and 5; **D**. Neuropodia of chaetiger 1, antero-dorsally; **E**. Chaetigers 5–9, dorsal view, beginning of branchiae and dorsal organs are seen; **F**. Posterior upper part of neuropodium of chaetiger 5; **G**. Neuropodium of chaetiger 9; **H**. Lower part of neuropodium of chaetiger 8; **I**. Modified spines of chaetiger 8; **J**. Thorax-abdomen junction, chaetigers 14–17; **K**. Neuropodium of chaetiger 10; **L**. Abdomen, dorsal view; **M**. Dorsal organ; **N**. Abdominal neuropodium; **O**. Forked chaeta from abdominal notopodium. Scale bars: 500 µm (**A, B**); 20 µm (**C, G, K, M**); 10 µm (**D, F, H, I**); 100 µm (**E**); 50 µm (**J**); 200 µm (**L**); 5 µm (**N**).

Body long, slender; with 119–226 chaetigers, 15–48 mm in length, 0.5–0.8 mm of thoracic width; thorax dorso-ventrally flattened, inflated in anterior part; abdomen cylindrical (Figs 12A, 13A). Color in ethanol whitish-yellow, chaetae brown. Thoracic chaetigers numbering 14–17. Prostomium conical, pointed, peristomium same length as chaetiger 1 (Figs 12B, 13B). Nuchal organs on anterior border of peristomium as transverse ciliated slits.

Branchiae starting from chaetiger 6, narrow triangular with tapering tips; gradually increasing in size, becoming equal in length or slightly longer than notopodia in abdomen (Figs 12B, 12D, 12F, 12G, 13B); with long cilia on both sides, not ciliated distally, ciliation continuing towards notopodia; no ciliation between branchiae (Fig. 13L).

Developed thoracic notopodial postchaetal lobes from chaetiger 2, digitiform; gradually increasing in size; in abdomen digitiform, equal in length or slightly shorter than branchiae (Fig. 12D, F, G). Thoracic neuropodial postchaetal lobes as low ridges, in posterior body region, one or two thoracic chaetigers with low triangular podal papilla in upper part of ridge (Figs 12C, 13C, 13J). Abdominal neuropodial lobes conical, with long digitiform outer lobe and reduced inner lobe with single thick projecting aciculum (Figs 12F, 12G, 13N). Parapodial flange not developed. Subpodal, stomach, and flange papillae absent.

Dorsal organs from chaetiger 6 round to oval ciliated spots (Fig. 13B, E, M). Ciliated lateral organs between bases of notopodia and neuropodia (Fig. 13C).

Notopodia bearing bundles of crenulated capillary chaetae in both thorax and abdomen; abdominal notopodia additionally having forked chaetae with subequal tines and pointed tips (Fig. 13O). Thoracic neuropodia with thick anteriormost row of uncini with membranous flap-like hoods in anterior row, three other rows of uncini and few capillaries in posteriormost row (Fig. 13C, D, G, J, K). In first chaetiger, anteriormost row slightly thicker than rest of uncini (Fig. 13D), in chaetigers 2–5 gradually enlarging (Fig. 13C), in other thoracic chaetigers anteriormost row two or three times thicker than rest of uncini, ~10 µm in diameter (Fig. 13G, I, K). In anterior thorax, five uncini forming anteriormost row, in posterior thorax their number decreasing to two uncini in uppermost part of neuropodia (Figs 12C, 12D, 12E, 13C, 13D, 13G, 13K). Uncini from second row slightly thicker and shorter than in posterior rows; hooded, slightly bent, with rounded conical tips, serrated with 8–10 ribs (Fig. 13C, F); fourth row of uncini short, developed only in ventral part, curved under anterior rows (Fig. 13C, H). In anterior thorax, five or six thin and long crenulated capillaries disposed in posterior row (Fig. 13C, D); in posterior thorax, capillaries decreasing in number to one or two and located dorsally (Fig. 13J, K). Abdominal neuropodia with bundle of capillary chaetae and single projecting thick straight aciculum; embedded part of aciculum slightly sigmoid, protruded part straight and pointed; similar to thoracic anteriormost uncini but not hooded (Figs 12F, 12G, 13N).

Pygidium with four short cirri around postero-dorsally located anal aperture.

###### Remarks.

*Leodamas gracilis* was described from Sri Lanka by Pillai (1961) and later reported from Vietnam by Gallardo (1968) and from Thailand by Eibye-Jacobsen (2002). Specimens studied here (Fig. 1, Lineage 5) were collected from the coasts of Oman and Kuwait (Failaka and Auha Islands). Although no genetic data are available for the specimens collected from the type locality, the morphology of the studied specimens agrees well with the paratype and the previous descriptions of *L. gracilis*.

*Leodamas gracilis* belongs to a group of species characterized by straight protruding abdominal aciculae (i.e., *L. brevithorax* (Eibye-Jacobsen, 2002), *L. cirratus* (Ehlers, 1897), *L. thalassae* (Amoureux, 1982), and *L. verax*) however, it is easily distinguishable from all of them by its unique anteriormost row of large uncini, up to two or three times thicker than the uncini in the other three rows.

###### Distribution.

Tambalagam Bay, Sri Lanka; Nha Trang, South Vietnam; coastal area of Thailand, at depths of 19–38 m. Arabian region: Oman and Kuwait, intertidal.

###### Habitat.

No habitat information was provided for Sri Lanka (Pillai 1961) and South Vietnam (Gallardo 1968) localities. In the coastal areas of Thailand, *L. gracilis* can be found in the subtidal zone (19–38 m), in sandy and muddy sediments, with or without shell fragments. In the Arabian region, the species can be found in the intertidal zone in muddy and sandy sediments.

## Discussion

Many annelid species with wide geographical ranges are known to be composed of species complexes, revealed by molecular methods but often also having subtle morphological differences becoming evident after a detailed examination of genetic lineages recovered by molecular analysis (e.g., Martin et al. 2020; Parapar et al. 2020; Radashevsky et al. 2020; Sikorski et al. 2021; Teixeira et al. 2022, 2023). Orbiniid species are not an exception, with cryptic speciation reported in *Leitoscoloplos pugettensis* (Pettibone, 1957), *Naineris laevigata* (Grube, 1855), *Naineris dendritica* (Kinberg, 1866), *Naineris setosa* (Verrill, 1900), *Orbiniella petersenae* Parapar, Moreira & Helgason, 2015, *Phylo foetida* (Claparède, 1868), *Scoloplos armiger* (Müller, 1776) (e.g., Kruse and Reise 2003; Kruse et al. 2003, 2004; Bleidorn et al. 2009; Carr et al. 2011; Zhadan et al. 2015; Álvarez and Budaeva 2023; Meca et al. 2024). In our study, we report that the widely distributed *Leodamas chevalieri* is a species complex composed of at least eleven putative species. The species name *Leodamas chevalieri* is assigned to the lineage found close to the type locality (Senegal). Despite some minor differences that could be considered intraspecific variation, the morphology of the studied specimens included in the molecular analysis generally agreed well with the redescribed type material. Until more molecular data are incorporated into the analysis, the species distribution is currently restricted to the coastal areas of Senegal, and all records from other regions are not considered to be *L. chevalieri*. *Leodamas gracilis* was found for the first time along the coast of Oman and Kuwait (Auha and Failaka islands), expanding its known distribution from Southeast Asia to the Arabian region. One of the lineages is described as a new species: *Leodamas edgari* sp. nov., distributed along the coast of Kuwait and Auha, Failaka, and Miscan islands. Further investigation is needed to describe and name the other discovered lineages, five in the Arabian region and three in western Africa.

We further clarify the delimitation of the group of *Leodamas* species inhabiting the east and south-east Atlantic, and the Indo-West Pacific which are very similar morphologically in bearing a single podal papilla in posteriormost thoracic chaetigers, five rows of uncini accompanied by few capillaries in the thoracic neuropodia, and a single, curved, protruding aciculum in the abdominal neuropodia. This group of species includes *L. australiensis* (from Australia), *L. chevalieri*, *L. dubius*, and *L. edgari* sp. nov. The species in this group can be separated by fine chaetal details (i.e., number of thoracic neuropodial capillaries, degree of curvature of abdominal projecting aciculae, type of forked chaetae, and hoods of thoracic uncini).

The descriptions provided by Gravier (1906) for *L. chevalieri* from the Red Sea and by Harmelin (1969) for *L. chevalieri candiensis* from the Mediterranean Sea were insufficient to evaluate the differences between both species. Similarly, the lack of information on the details of chaetal morphology in *L. australiensis* from the Mediterranean Sea (Sala-Mirete et al. 2023) did not allow clarification of the status of this population despite a strong difference in the 16S marker. The taxonomic statuses of *L. australiensis* (from the Mediterranean Sea), *L. chevalieri* (from the Red Sea), and *L. chevalieri candiensis* require clarification by the inclusion of genetic data with a morphological revision of the three species. To initiate this revision, we here provide an overview of the main morphological characters in all the species discussed in the present study (Table 2). A key to the species with unambiguous taxonomic status is provided below.

**Table 2.** Discriminatory characters among *Leodamas edgari* sp. nov., *L. chevalieri candiensis*, *L. dubius*, *L. gracilis*, and the different populations of *L. chevalieri* and *L. australiensis*. *We believe the fifth row of thoracic uncini was overlooked by researchers since it is only developed in the ventral part of neuropodia. Abbreviations: TCH, thoracic chaetiger.

	*L. edgari* sp. nov.	*L. chevalieri*	*L. chevalieri*	*L. chevalieri candiensis*	*L. australiensis*	*L. australiensis*	*L. dubius*	*L. gracilis*
**Source**	Present study; type material	Present study; type material	Gravier (1906)	Harmelin (1969)	Zhadan et al. (2015); Zhadan (2020)	Sala-Mirete et al. (2023)	Tebble (1955); own observations of type material	Present study; Pillai (1961); Gallardo (1968); Eibye-Jacobsen (2002)
**Locality**	Kuwait (Arabian Gulf)	Senegal (type locality)	Red Sea	Mediterranean	Australia	Mediterranean	Ghana (West Africa)	SE Asia and Arabian region
**Thoracic chaetigers number**	16–21	18–23 (21–22 in Fauvel)	19–26	21	18–23	19–27	21–23	13–17 (mostly 15 in Arabian region)
**Branchiae start**	6	6	6	6	6	6	7	6
**Forked chaetae**	Present, with unequal tines	Present, with subequal tines	Present, 2 or 3, with unequal tines	Absent in holotype but present in paratypes. Unequal tines	Present, with subequal tines.	1 or 2 forked chaetae, with smooth shaft and subequal tines	Absent?	Present, with subequal tines
**Notoacicula**	Present, 2, thin	Present, 2, thin	?	Aciculae are replaced by bristles with a robust shaft	Present, 1–3, thin	Present, 2, thin	Present	Present, 2, thin
**Abdominal neuropodial acicula**	Strongly hooked in some segments	Almost straight or slightly curved	Two aciculae in anterior abdomen; in posterior only one very prominent aciculum with a tip curved ventrally	Straight, slightly curved	Strongly hooked in some segments	From nearly straight to curved 180°	Strongly hooked in some segments	Straight
**Capillaries in thoracic neuropodia**	1–3 thin disposed dorsally	In all TCH, 1 in middle thorax, up to 3 in anterior and posterior segments (2–3 in Fauvel)	2 or 3 in upper part	8 or 9 very fine in the upper part	1–4 thin, more in anterior and posterior segments	absent?	4–8, forming half a row in dorsal part, comparatively big	5 or 6 thin and long disposed in posterior row; 1 or 2 in posterior thorax and disposed dorsally
**Rows of thoracic uncini**	5	5	4*	4*	5	5	5	4
**Thoracic uncini**	Some with membranous hood. Smooth in anterior row, slightly or clearly serrated with up to 7 ribs in posterior row.	All with membranous hood. In type specimens, mostly smooth; in non-type specimens, mostly serrated with up to 9 ribs.	All with membranous hood and serrated with 7 or 8 ribs. Only 3 rows of uncini in the first two TCH, 4 from the 3^rd^.	All with membranous hood. Clearly serrated.	Very thick hoods in upper posterior uncini from posterior thorax.	No mention on hoods and serration.	In posterior thorax anterior row of very thick uncini. Serration or hoods unknown	Anteriormost row up to 2–3 times thicker than the other 3. Uncini from 2^nd^ row with membranous hood and serrated with 8–10 ribs.
**Anal cirri**	4 short, equal in length	?	4 medium, equal in length	?	4 short	?	Dorsal much longer than ventral	4 short, equal in length

### Key to species of *Leodamas* discussed in this study from the East and South-East Atlantic and the Indo-West Pacific

**Table:** 

1	Branchiae starting at 7^th^ chaetiger	***L. dubius* (Tebble, 1955)**
–	Branchiae starting at 6^th^ chaetiger	**2**
2	Straight aciculum in abdominal neuropodia, very thick straight spines in the first row of thoracic neuropodia	***L. gracilis* (Pillai, 1961)**
–	Aciculum in abdominal neuropodia slightly or strongly curved, first row of uncini is similar with subsequent rows	**3**
3	Slightly curved aciculum in abdominal neuropodia	***L. chevalieri* (Fauvel, 1902) (Senegal)**
–	Strongly hooked aciculum in some abdominal neuropodia	**4**
4	Thoracic uncini with membranous hoods	***L. edgari* sp. nov**.
–	Thoracic uncini (posteriorly) with very thick hoods	***L. australiensis* (Hartmann-Schröder, 1979) (Australia)**

## Supplementary Material

XML Treatment for Leodamas

XML Treatment for Leodamas chevalieri

XML Treatment for Leodamas edgari

XML Treatment for Leodamas gracilis

## Additional information

### Use of AI

No use of AI was reported.

### Author ORCIDs

Miguel A. Meca https://orcid.org/0000-0002-3798-6809

Anna Zhadan https://orcid.org/0000-0001-6560-3732

Manal Al-Kandari https://orcid.org/0000-0003-0073-7929

Nataliya Budaeva https://orcid.org/0000-0001-9748-2285

### Data availability

All of the data that support the findings of this study are available in the main text or Supplementary Information.

## Supplementary materials

Supplementary material 1 Sampling data and genetic distances Data type xlsx Explanation note **table SS1**. List of material used in this study with sampling information, museum numbers, voucher numbers, GenBank accession numbers and BOLD process IDs; **table SS2**. Pairwise genetic distances within and between *Leodamas* lineages from Arabian and western African regions. https://binary.pensoft.net/file/1582170This dataset is made available under the Open Database License (http://opendatacommons.org/licenses/odbl/1.0/). The Open Database License (ODbL) is a license agreement intended to allow users to freely share, modify, and use this Dataset while maintaining this same freedom for others, provided that the original source and author(s) are credited. Miguel A. Meca, Anna Zhadan, Manal Al-Kandari, Nataliya Budaeva

Supplementary material 2 Phylogenetic trees regarding COI and ITS2 markers, and species delimitation results Data type docx Explanation note **fig. S1**. Maximum likelihood (ML) analysis based on the concatenated dataset of COI and ITS2. Bootstrap values are shown on the nodes. **fig. S2**. Maximum likelihood (ML) analysis based on COI marker. Bootstrap values are shown on the nodes. **fig. S3**. Bayesian inference (BI) based on COI marker. Bayesian posterior probabilities are shown on the nodes. **fig. S4**. Maximum likelihood (ML) analysis based on ITS2 marker. Bootstrap values are shown on the nodes. **fig. S5**. Bayesian inference (BI) based on ITS2 marker. Bayesian posterior probabilities are shown on the nodes. **file S1**. PTP species delimitation. **A**. COI. **B**. ITS2. **A**. Species delimitation – COI fragment: PTP results. **B**. Species delimitation – ITS2 fragment: PTP results. **file S2**. ASAP species delimitation. **A**. COI. **B**. ITS2. **A**. Species delimitation – COI fragment: ASAP results. **B**. Species delimitation – ITS2 fragment: ASAP results. https://binary.pensoft.net/file/1582171This dataset is made available under the Open Database License (http://opendatacommons.org/licenses/odbl/1.0/). The Open Database License (ODbL) is a license agreement intended to allow users to freely share, modify, and use this Dataset while maintaining this same freedom for others, provided that the original source and author(s) are credited. Miguel A. Meca, Anna Zhadan, Manal Al-Kandari, Nataliya Budaeva

## References

[B1] Al-Kandari M, Sattari Z, Hussain S, Radashevsky VI, Zhadan A (2019) Checklist of intertidal polychaetes (Annelida) of Kuwait, Northern part of the Arabian Gulf. Regional Studies in Marine Science 32: 100872. 10.1016/j.rsma.2019.100872

[B2] Álvarez R, Budaeva N (2023) How complex is the *Naineris setosa* species complex? First integrative study of a presumed cosmopolitan and invasive annelid (Sedentaria: Orbiniidae). Zootaxa 5375(3): 349–378. 10.11646/zootaxa.5375.3.3 38220815

[B3] Amoureux L (1976) Annélides polychètes récoltés par J. Stirn en 1969, sur les côtes Marocaines du Détroit de Gibraltar. Cuadernos de Ciencias Biológicas. Universidad de Granada 5: 5–33. 10.5962/p.280636

[B4] Blake JA (2000) A new genus and species of polychaete worm (Family Orbiniidae) from methane seeps in the Gulf of Mexico, interrelationships of the genera of Orbiniidae. Cahiers de Biologie Marine 41(4): 435–449. 10.21411/CBM.A.84F1D61E

[B5] Blake JA (2017) PolychaetaOrbiniidae from antarctica, the Southern Ocean, the abyssal Pacific ocean, and off South America. Zootaxa 4218(1): 1–145. 10.11646/zootaxa.4218.1.1 28187682

[B6] Blake JA (2020) New species and records of deep-water Orbiniidae (Annelida, Polychaeta) from the Eastern Pacific continental slope, abyssal Pacific Ocean, and the South China Sea. Zootaxa 4730(1): 1–61. 10.11646/zootaxa.4730.1.1 32229835

[B7] Blake JA (2021) New species and records of Orbiniidae (Annelida, Polychaeta) from continental shelf and slope depths of the Western North Atlantic Ocean. Zootaxa 4930(1): 1–123. 10.11646/zootaxa.4930.1.1 33756810

[B8] Bleidorn C, Hill N, Erséus C, Tiedemann R (2009) On the role of character loss in orbiniid phylogeny (Annelida): Molecules vs. morphology. Molecular Phylogenetics and Evolution 52(1): 57–69. 10.1016/j.ympev.2009.03.022 19345272

[B9] Budaeva N, Agne S, Ribeiro PA, Straube N, Preick M, Hofreiter M (2024) Wide-spread dispersal in a deep-sea brooding polychaete: The role of natural history collections in assessing the distribution in quill worms (Onuphidae, Annelida). Frontiers in Zoology 21(1): 1. 10.1186/s12983-023-00520-0 PMC10795374 38233869

[B10] Carr CM, Hardy SM, Brown TM, Macdonald TA, Hebert PD (2011) A tri-oceanic perspective: DNA barcoding reveals geographic structure and cryptic diversity in Canadian polychaetes. PLoS One 6(7): e22232. 10.1371/journal.pone.0022232 PMC3136506 21829451

[B11] Claparède E (1868) Les annélides chétopodes du Golfe de Naples. Mémoires de la Société de Physique et d'Histoire Naturelle de Genève 19(2): 313–584.

[B12] Day JH (1954) The Polychaeta of Tristan da Cunha 1937–1938. Results of the Norwegian Expedition to Tristan da Cunha 1937–1938 29: 1–35.

[B13] Day JH (1973) New Polychaeta from Beaufort, with a key to all species recorded from North Carolina. NOAA Technical Report. National Marine Fisheries Circular 375: 1–140. 10.5962/bhl.title.62852

[B14] Ehlers E (1897) Polychaeten. Ergebnisse der Hamburger Magalhaensischen Sammelreise 3: 1–148.

[B15] Eibye-Jacobsen D (2002) The Orbiniidae (Annelida: Polychaeta) of the BIOSHELF Project, Andaman Sea, Thailand. Phuket Marine Biological Center Special Publication 24: 77–99.

[B16] Fauvel P (1902) Annélides Polychètes de la Casamance rapportées par M. Aug. Chevalier. Bulletin de la Société Linnéenne de Normandie 5: 59–105.

[B17] Fauvel P (1919) Annélides Polychètes de Madagascar, de Djibouti, et du Golfe Persique. Archives de Zoologie Expérimentale et Générale 58: 315–473. 10.5962/bhl.part.8154

[B18] Fauvel P (1953) AnnelidaPolychaeta. The fauna of India, including Pakistan, Ceylon, Burma and Malaya. The Indian Press, Allahabad, 507 pp.

[B19] Gallardo VA (1968) Polychaeta from the bay of Nha Trang, south Viet Nam. Naga Report 4(3): 35–279.

[B20] Gravier C (1906) Sur les Annélides Polychètes recueillies par l'Expédition antarctique française (Hésioniens, Phyllodociens, Néréidiens, Euniciens). Bulletin du Musée D'histoire Naturelle 12(6): 386–391. 10.5962/p.328647

[B21] Grube AE (1855) Beschreibungen neuer oder wenig bekannter Anneliden. Archiv für Naturgeschichte 21: 81–128. 10.5962/bhl.part.13989

[B22] Harmelin JG (1969) Contribution à l’étude de l’endofaune des prairies *d’Halophila stipulacea* de Méditerranée orientale. Recueil des Travaux de la Station marine Endoume 45(61): 305–316.

[B23] Hartman O (1942) A review of the types of polychaetous annelids at the Peabody Museum of Natural History, Yale University. Bulletin of the Bingham Oceanographic Collection 8: 1–98.

[B24] Hartman O (1957) Orbiniidae, Apistobranchidae, Paraonidae and Longosomidae. Allan Hancock Pacific Expeditions 15(3): 211–393. 10.5281/zenodo.16175719

[B25] Hartmann-Schröder G (1979) Die Polychaeten der tropischen Nordwestküste Australiens (zwischen Derby im Norden und Port Hedland im Süden). In: Hartmann-Schröder G and Hartmann G (Eds) Zur Kenntnis des Eulitorals der australischen Küsten un- ter besonder Berücksichtigung der Polychaeten und Ostracoden (Teil 2 und Teil 3). Mitteilungen aus dem Hamburgischen zoologischen Museum und Institut 76: 77–218.

[B26] Hutchings P, Kupriyanova E (2018) Cosmopolitan polychaetes–fact or fiction? Personal and historical perspectives. Invertebrate Systematics 32(1): 1–9. 10.1071/IS17035

[B27] Katoh K, Standley DM (2013) MAFFT multiple sequence alignment software version 7: Improvements in performance and usability. Molecular Biology and Evolution 30(4): 772–780. 10.1093/molbev/mst010 PMC3603318 23329690

[B28] Kearse M, Moir R, Wilson A, Stones-Havas S, Cheung M, Sturrock S, Buxton S, Cooper A, Markowitz S, Duran C, Thierer T, Ashton B, Meintjes P, Drummond A (2012) Geneious Basic: An integrated and extendable desktop software platform for the organization and analysis of sequence data. Bioinformatics (Oxford, England) 28(12): 1647–1649. 10.1093/bioinformatics/bts199 PMC3371832 22543367

[B29] Kinberg J (1866) Annulata nova. Öfversight af Kungliga Vetenskaps–Adakemiens Förhandlingar, Stockholm 22, 239–258.

[B30] Kruse I, Reise K (2003) Reproductive isolation between intertidal and subtidal Scoloplos armiger (Polychaeta, Orbiniidae) indicates sibling species in the North Sea. Marine Biology 143(3): 511–517. 10.1007/s00227-003-1112-x

[B31] Kruse I, Reusch TB, Schneider MV (2003) Sibling species or poecilogony in the polychaete *Scoloplos armiger*? Marine Biology 142(5): 937–947. 10.1007/s00227-002-1007-2

[B32] Kruse I, Strasser M, Thiermann F (2004) The role of ecological divergence in speciation between intertidal and subtidal *Scoloplos armiger* (Polychaeta, Orbiniidae). Journal of Sea Research 51(1): 53–62. 10.1016/j.seares.2003.05.004

[B33] Kumar S, Stecher G, Li M, Knyaz C, Tamura K (2018) MEGA X: Molecular evolutionary genetics analysis across computing platforms. Molecular Biology and Evolution 35(6): 1547–1549. 10.1093/molbev/msy096 PMC5967553 29722887

[B34] Lanfear R, Frandsen PB, Wright AM, Senfeld T, Calcott B (2017) PartitionFinder 2: New methods for selecting partitioned models of evolution for molecular and morphological phylogenetic analyses. Molecular Biology and Evolution 34(3): 772–773. 10.1093/molbev/msw260 28013191

[B35] Martin D, Gil J, Zanol J, Meca MA, Pérez Portela R (2020) Digging the diversity of Iberian bait worms *Marphysa* (Annelida, Eunicidae). PLoS One 15(1): e0226749. 10.1371/journal.pone.0226749 PMC6975537 31967996

[B36] Meca MA (2025) Systematic revision of Orbiniidae (Annelida). PhD thesis, University Museum of Bergen, University of Bergen, Norway. https://hdl.handle.net/11250/3175624

[B37] Meca MA, Zhadan A, Struck TH (2021) The Early Branching Group of Orbiniida Sensu Struck et al., 2015: Parergodrilidae and Orbiniidae. Diversity 13(1): 29. 10.3390/d13010029

[B38] Meca MA, Kongsrud JA, Kongshavn K, Alvestad T, Meißner K, Budaeva N (2024) Diversity of *Orbiniella* (Orbiniidae, Annelida) in the North Atlantic and the Arctic. ZooKeys 1205: 51–88. 10.3897/zookeys.1205.120300 PMC11211660 38947165

[B39] Meißner K, Schwentner M, Götting M, Knebelsberger T, Fiege D (2023) Polychaetes distributed across oceans – Examples of widely recorded species from abyssal depths of the Atlantic and Pacific Oceans. Zoological Journal of the Linnean Society 199(4): 906–944. 10.1093/zoolinnean/zlad069

[B40] Meyer A, Bleidorn C, Rouse GW, Hausen H (2008) Morphological and molecular data suggest a cosmopolitan distribution of the polychaete *Proscoloplos cygnochaetus* Day, 1954 (Annelida, Orbiniidae). Marine Biology 153(5): 879–889. 10.1007/s00227-007-0860-4

[B41] Miller MA, Pfeiffer W, Schwartz T (2012) The CIPRES science gateway: enabling high-impact science for phylogenetics researchers with limited resources. In: Proceedings of the 1^st^ Conference of the Extreme Science and Engineering Discovery Environment: Bridging from the extreme to the campus and beyond, Chicago Illinois (USA), July 2012. Association for Computing Machinery, New York, 1–8. 10.1145/2335755.2335836

[B42] Müller OF (1776) Zoologiae Danicae Prodromus seu Animalium Daniae et Norvegiae indigenarum characteres, nomina, et synonyma imprimis popularium. Typiis Hallageriis, Hafniae, Copenhagen, 282 pp. 10.5962/bhl.title.13268

[B43] Nguyen LT, Schmidt HA, Von Haeseler A, Minh BQ (2015) IQ-TREE: A fast and effective stochastic algorithm for estimating maximum-likelihood phylogenies. Molecular Biology and Evolution 32(1): 268–274. 10.1093/molbev/msu300 PMC4271533 25371430

[B44] Nygren A, Eklöf J, Pleijel F (2009) Arctic-boreal sibling species of *Paranaitis* (Polychaeta, Phyllodocidae). Marine Biology Research 5(4): 315–327. 10.1080/17451000802441301

[B45] Ong B (1995) Polychaetes of Telok-Aling, Penang, Malaysia. The Raffles Bulletin of Zoology 43(1): 257–283.

[B46] Parapar J, Moreira J, Helgason GV (2015) First record of genus *Orbiniella* Day, 1954 (Polychaeta: Orbiniidae) in North Atlantic Ocean with the description of a new species. Zootaxa 4006(2): 330–346. 10.11646/zootaxa.4006.2.5 26623770

[B47] Parapar J, Capa M, Nygren A, Moreira J (2020) To name but a few: Descriptions of five new species of *Terebellides* (Annelida, Trichobranchidae) from the North East Atlantic. ZooKeys 992: 1–58. 10.3897/zookeys.992.55977 PMC7677295 33223905

[B48] Pettibone MH (1957) North American genera of the family Orbiniidae (Annelida: Polychaeta), with descriptions of new species. Journal of the Washington Academy of Sciences 47(5): 159–167.

[B49] Pillai TG (1961) AnnelidaPolychaeta of Tambalagam Lake, Ceylon. Ceylon Journal of Science. Biological Sciences 4(1): 1–40.

[B50] Puillandre N, Brouillet S, Achaz G (2021) ASAP: Assemble species by automatic partitioning. Molecular Ecology Resources 21(2): 609–620. 10.1111/1755-0998.13281 33058550

[B51] Radashevsky VI, Pankova VV, Malyar VV, Neretina TV, Choi JW, Yum S, Houbin C (2020) Molecular analysis of *Spiophanes bombyx* complex (Annelida: Spionidae) with description of a new species. PLoS One 15(7): e0234238. 10.1371/journal.pone.0234238 PMC7329067 32609771

[B52] Rambaut A, Drummond AJ, Xie D, Baele G, Suchard MA (2018) Posterior summarization in Bayesian phylogenetics using Tracer 1.7. Systematic Biology 67(5): 901–904. 10.1093/sysbio/syy032 PMC6101584 29718447

[B53] Ronquist F, Teslenko M, Van Der Mark P, Ayres DL, Darling A, Höhna S, Larget B, Liu L, Suchard MA, Huelsenbeck JP (2012) MrBayes 3.2: Efficient Bayesian phylogenetic inference and model choice across a large model space. Systematic Biology 61(3): 539–542. 10.1093/sysbio/sys029 PMC3329765 22357727

[B54] Sala-Mirete A, López E, Fernández-Alías A, Sánchez-Fernández O, Marcos C, Pérez-Ruzafa A (2023) *Leodamas australiensis* (Hartmann-Schröder, 1979) (Polychaeta, Orbiniidae), a new alien species in the Mediterranean, and its ecology in the Mar Menor coastal lagoon (SE Spain). BioInvasions Records 12(4): 993–1013. 10.3391/bir.2023.12.4.13

[B55] Sikorski AV, Radashevsky VI, Castelli A, Pavlova LV, Nygren A, Malyar VV, Borisova PB, Mikac B, Rousou M, Martin D, Gil J, Pacciardi L, Langeneck J (2021) Revision of the *Laonice bahusiensis* complex (Annelida: Spionidae) with a description of three new species. Zootaxa 4996(2): 253–283. 10.11646/zootaxa.4996.2.2 34810532

[B56] Sun Y, Sui J, Li X (2018) A new species of *Leodamas* Kinberg, 1866 (Polychaeta: Orbiniidae) from the Yellow Sea and the East China Sea. Acta Oceanologica Sinica 37(10): 130–135. 10.1007/s13131-018-1313-2

[B57] Tebble N (1955) The polychaete fauna of the Gold Coast [=Ghana]. Bulletin of the British Museum (Natural History). Series Zoology 3: 59–148.

[B58] Teixeira MA, Bakken T, Vieira PE, Langeneck J, Sampieri BR, Kasapidis P, Ravara A, Nygren A, Costa FO (2022) The curious and intricate case of the European *Hediste diversicolor* (Annelida, Nereididae) species complex, with description of two new species. Systematics and Biodiversity 20(1): 1–39. 10.1080/14772000.2022.2116124

[B59] Teixeira MA, Vieira PE, Fenwick D, Langeneck J, Pleijel F, Sampieri BR, Hernández JC, Ravara A, Costa FO, Nygren A (2023) Revealing the diversity of the green Eulalia (Annelida, Phyllodocidae) species complex along the European coast, with description of three new species. Organisms, Diversity & Evolution 23(3): 477–503. 10.1007/s13127-022-00597-1

[B60] Vân Le HL, Lecointre G, Perasso R (1993) A 28S rRNA-based phylogeny of the gnathostomes: First steps in the analysis of conflict and congruence with morphologically based cladograms. Molecular Phylogenetics and Evolution 2(1): 31–51. 10.1006/mpev.1993.1005 8081546

[B61] Verrill AE (1900) Additions to the Turbellaria, Nemertina, and Annelida of the Bermudas, with revisions of some New England genera and species. Transactions of the Connecticut Academy of Arts and Sciences 10(2): 595–671. 10.5962/bhl.part.7035

[B62] Wehe T, Fiege D (2002) Annotated checklist of the polychaete species of the seas surrounding the Arabian Peninsula: Red Sea, Gulf of Aden, Arabian Sea, Gulf of Oman, Arabian Gulf. Fauna of Arabia 19: 7–238.

[B63] Wesenberg-Lund E (1949) Polychaetes of the Iranian Gulf. Danish Scientific Investigations in Iran 4: 247–400.

[B64] Zhadan A (2020) Review of Orbiniidae (Annelida, Sedentaria) from Australia. Zootaxa 4860(4): 451–502. 10.11646/zootaxa.4860.4.1 33055877

[B65] Zhadan A, Stupnikova A, Neretina T (2015) Orbiniidae (Annelida: Errantia) from Lizard Island, Great Barrier Reef, Australia with notes on orbiniid phylogeny. Zootaxa 4019(1): 773–801. 10.11646/zootaxa.4019.1.27 26624087

[B66] Zhang J, Kapli P, Pavlidis P, Stamatakis A (2013) A general species delimitation method with applications to phylogenetic placements. Bioinformatics (Oxford, England) 29(22): 2869–2876. 10.1093/bioinformatics/btt499 PMC3810850 23990417

