# Supplementary material for: The widespread Leodamas chevalieri (Annelida, Orbiniidae) revealed as a species complex, with descriptions of a new species and a new record for the Arabian region
Source: Zookeys. 2026 Apr 3;1276:61–94. doi: 10.3897/zookeys.1276.173213 (PMC13069388; doi:10.3897/zookeys.1276.173213)
Supplement: Supplementary material 2 — Phylogenetic trees regarding COI and ITS2 markers, and species delimitation results [file zookeys-1276-061_article-173213__-s002.docx]

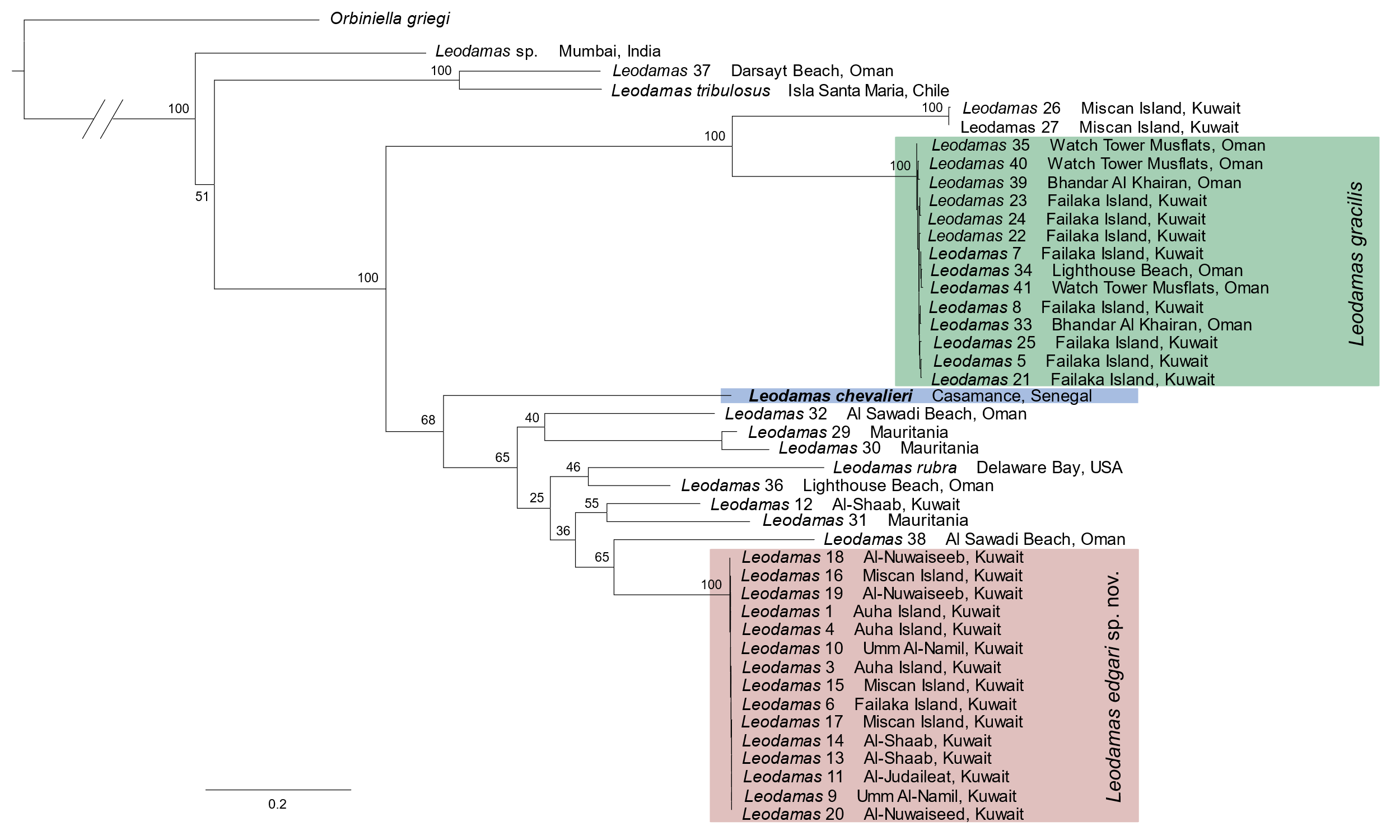


**Fig. S1.** Maximum likelihood (ML) analysis based on the concatenated dataset of COI and ITS2. Bootstrap values are shown on the nodes.


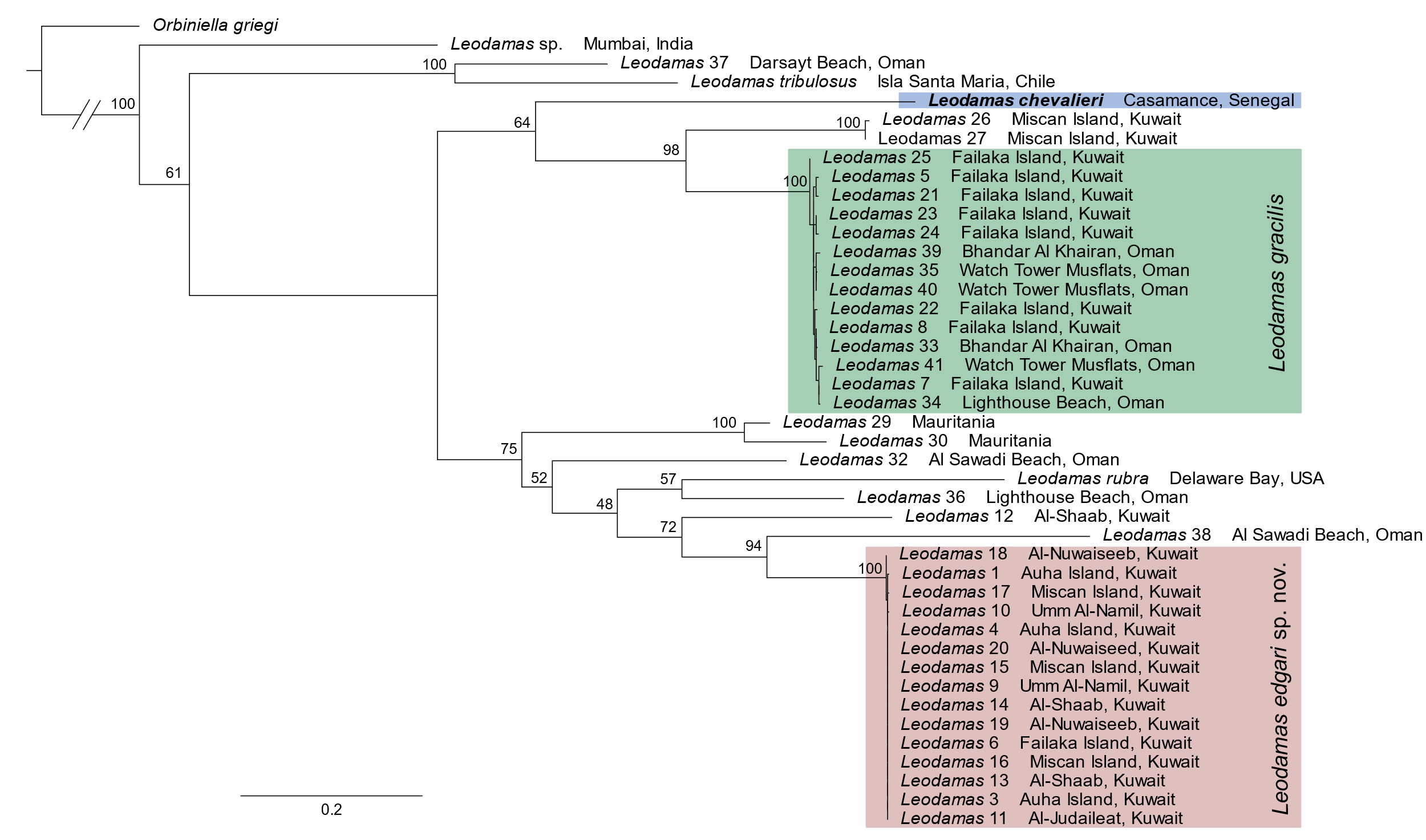


**Fig. S2.** Maximum likelihood (ML) analysis based on COI marker. Bootstrap values are shown on the nodes.

**
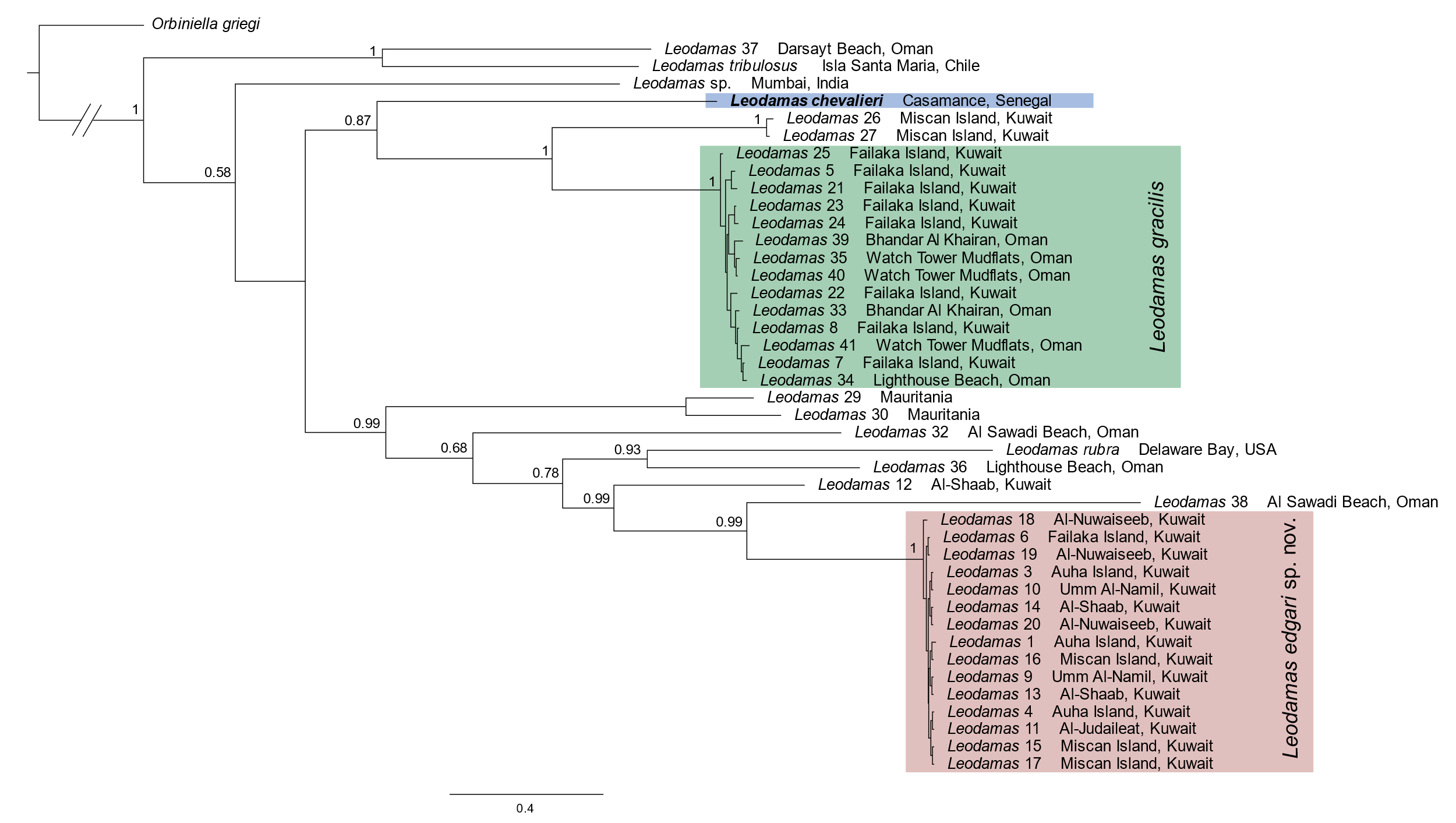
**

**Fig. S3.** Bayesian inference (BI) based on COI marker. Bayesian posterior probabilities are shown on the nodes.

**
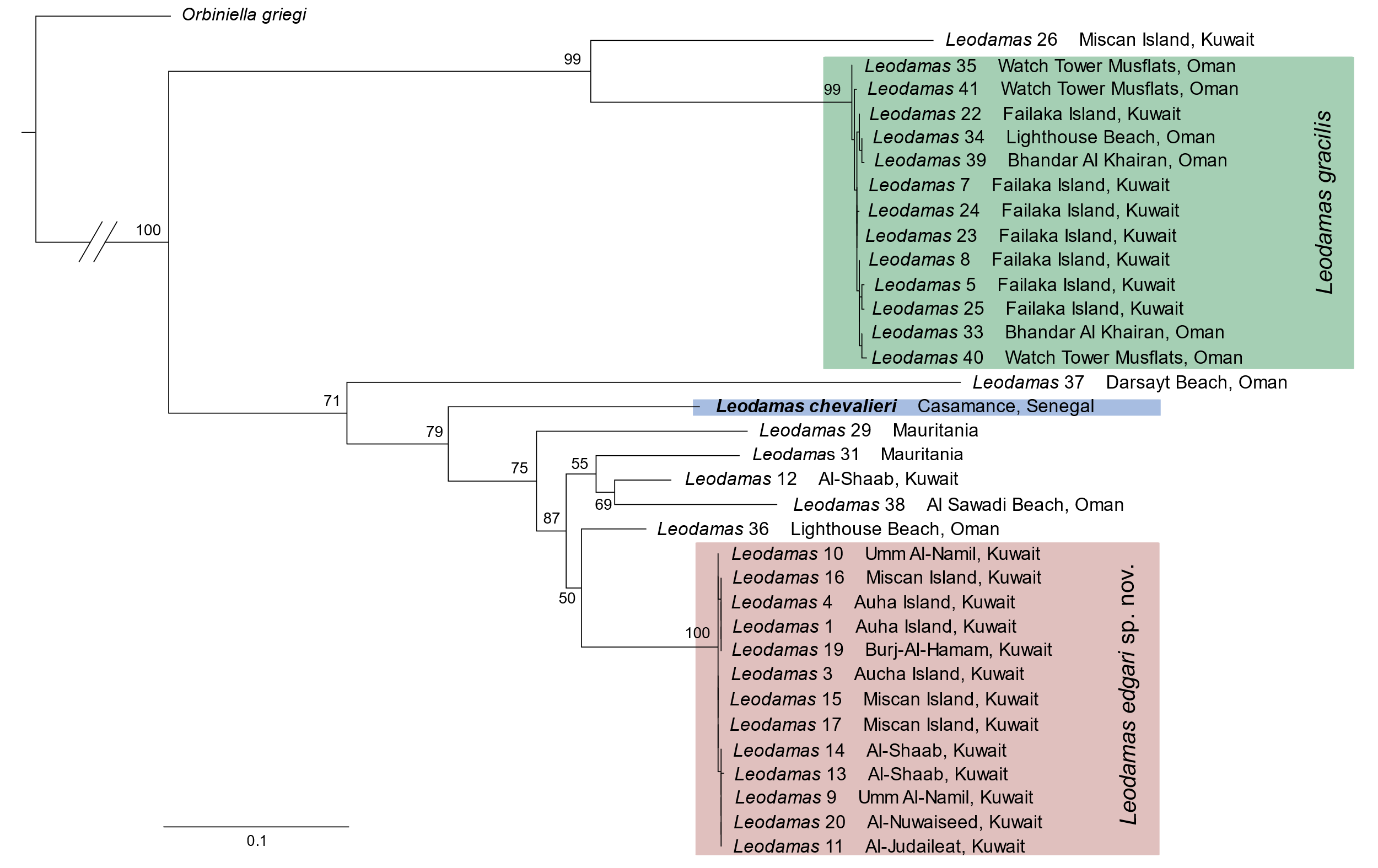
**

**Fig. S4.** Maximum likelihood (ML) analysis based on ITS2 marker. Bootstrap values are shown on the nodes.

**
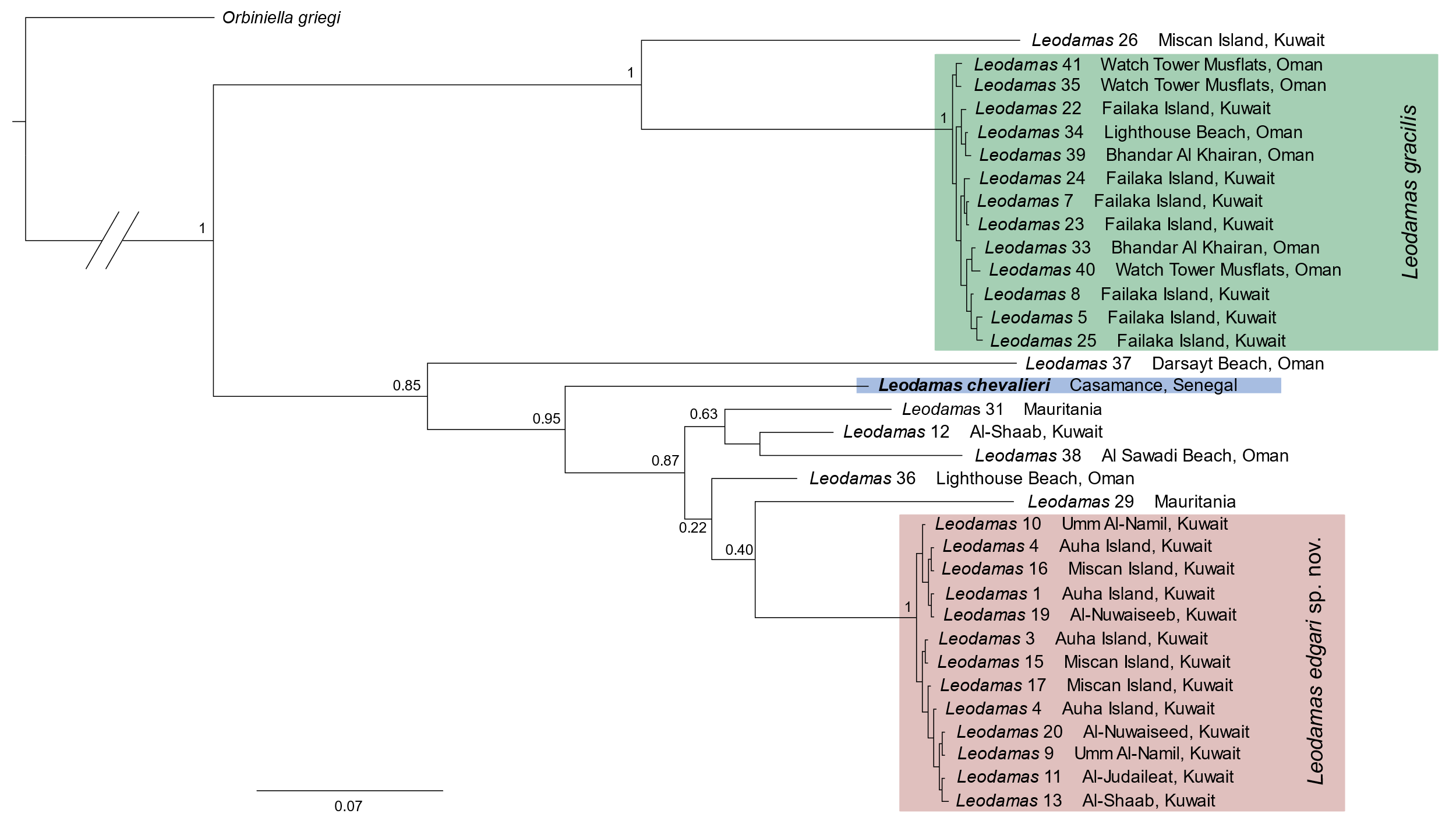
**

**Fig. S5.** Bayesian inference (BI) based on ITS2 marker. Bayesian posterior probabilities are shown on the nodes.

**File S1.** PTP species delimitation. A. COI. B. ITS2.

**A. Species delimitation – COI fragment: PTP results**

**Results based on the Maximum Likelihood reconstruction**

**Maximum Likelihood tree**


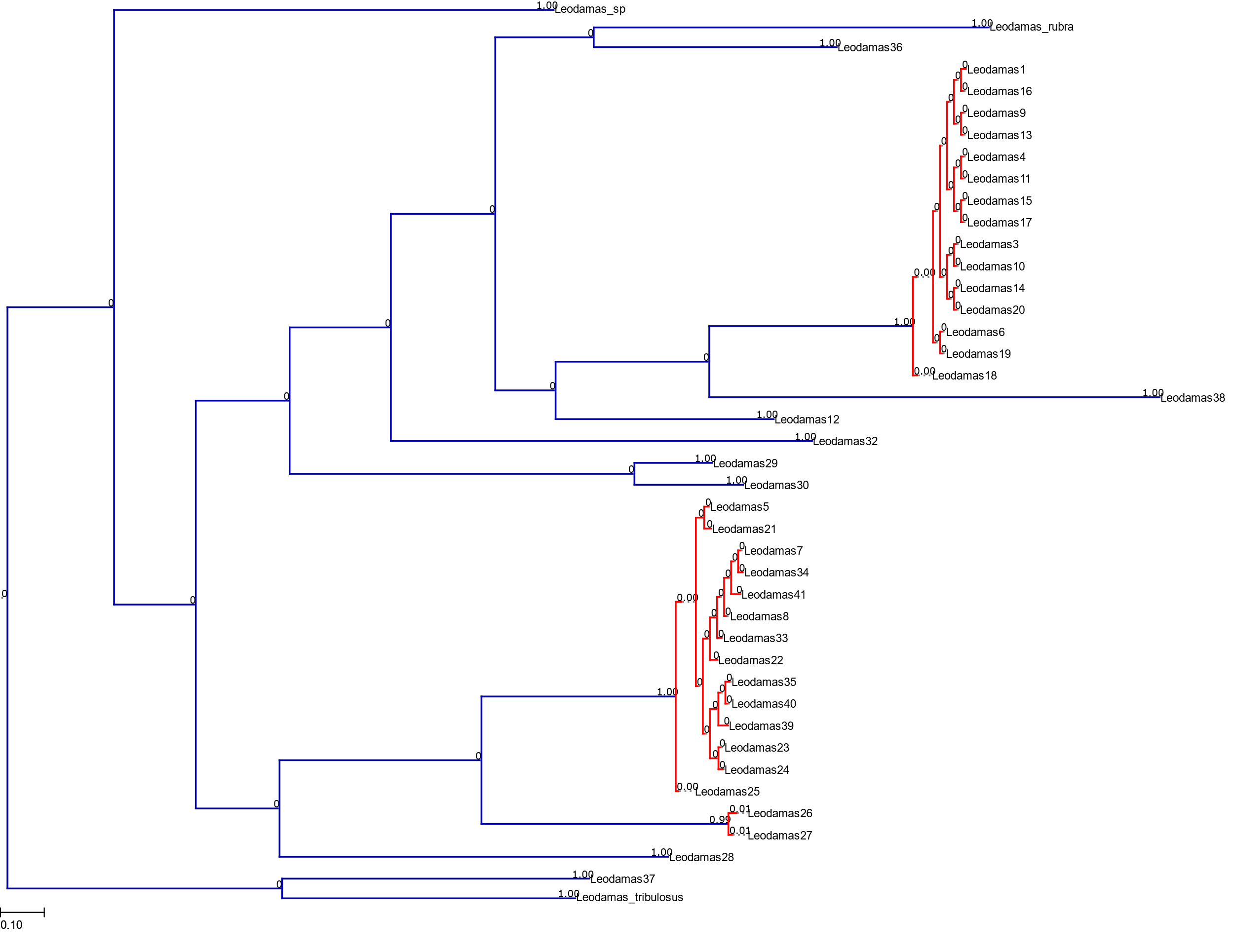


**Maximum Likelihood partition**

*Leodamas edgari* sp. nov. (support = 0.998)

Leodamas1,Leodamas16,Leodamas9,Leodamas13,Leodamas4,Leodamas11,Leodamas15,Leodamas17,Leodamas3,Leodamas10,Leodamas14,Leodamas20,Leodamas6,Leodamas19,Leodamas18

Lineage 14 (support = 1.000)

Leodamas38

Lineage 13 (support = 1.000)

Leodamas12

Lineage 7 (support = 1.000)

Leodamas32

Lineage 1 (support = 1.000)

Leodamas_sp

Lineage 11 (support = 1.000)

Leodamas_rubra

Lineage 10 (support = 1.000)

Leodamas36

*Leodamas chevalieri* (support = 1.000)

Leodamas28

Lineage 2 (support = 1.000)

Leodamas37

Lineage 3 (support = 1.000)

Leodamas_tribulosus

*Leodamas gracilis* (support = 0.999)

Leodamas5,Leodamas21,Leodamas7,Leodamas34,Leodamas41,Leodamas8,Leodamas33,Leodamas22,Leodamas35,Leodamas40,Leodamas39,Leodamas23,Leodamas24,Leodamas25

Lineage 4 (support = 0.990)

Leodamas26,Leodamas27

Lineage 8 (support = 1.000)

Leodamas29

Lineage 9 (support = 1.000)

Leodamas30

**Results based on the Bayesian inference reconstruction**

**Bayesian inference tree**


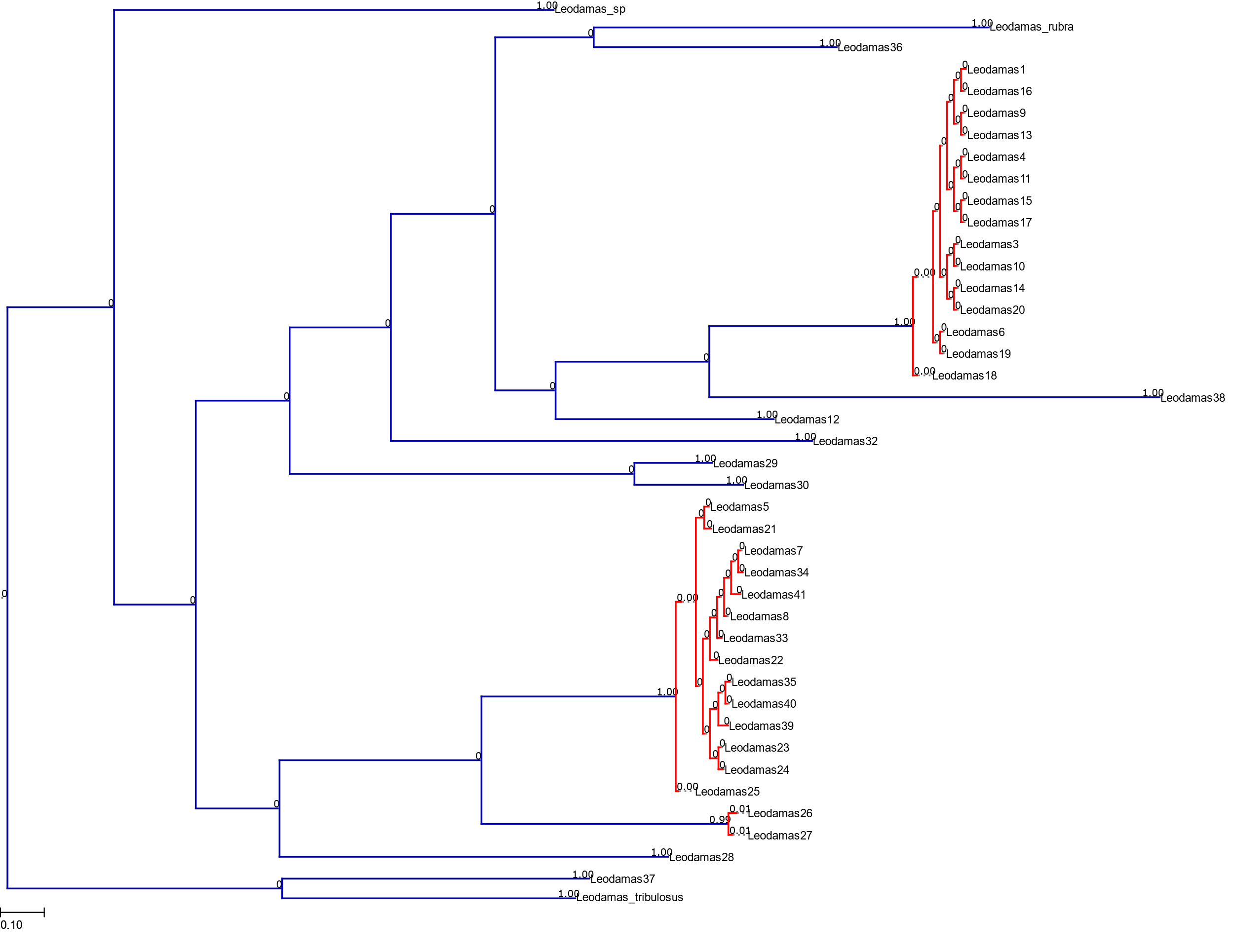


**Most supported partition found by simple heuristic search**

*Leodamas edgari* sp. nov. (support = 0.998)

Leodamas1,Leodamas16,Leodamas9,Leodamas13,Leodamas4,Leodamas11,Leodamas15,Leodamas17,Leodamas3,Leodamas10,Leodamas14,Leodamas20,Leodamas6,Leodamas19,Leodamas18

Lineage 14 (support = 1.000)

Leodamas38

Lineage 13 (support = 1.000)

Leodamas12

Lineage 7 (support = 1.000)

Leodamas32

Lineage 1 (support = 1.000)

Leodamas_sp

Lineage 11 (support = 1.000)

Leodamas_rubra

Lineage 10 (support = 1.000)

Leodamas36

*Leodamas chevalieri* (support = 1.000)

Leodamas28

Lineage 2 (support = 1.000)

Leodamas37

Lineage 3 (support = 1.000)

Leodamas_tribulosus

*Leodamas gracilis* (support = 0.999)

Leodamas5,Leodamas21,Leodamas7,Leodamas34,Leodamas41,Leodamas8,Leodamas33,Leodamas22,Leodamas35,Leodamas40,Leodamas39,Leodamas23,Leodamas24,Leodamas25

Lineage 4 (support = 0.990)

Leodamas26,Leodamas27

Lineage 8 (support = 1.000)

Leodamas29

Lineage 9 (support = 1.000)

Leodamas30

**B. Species delimitation – ITS2 fragment: PTP results**

**Results based on the Maximum Likelihood reconstruction**

**Maximum Likelihood tree**


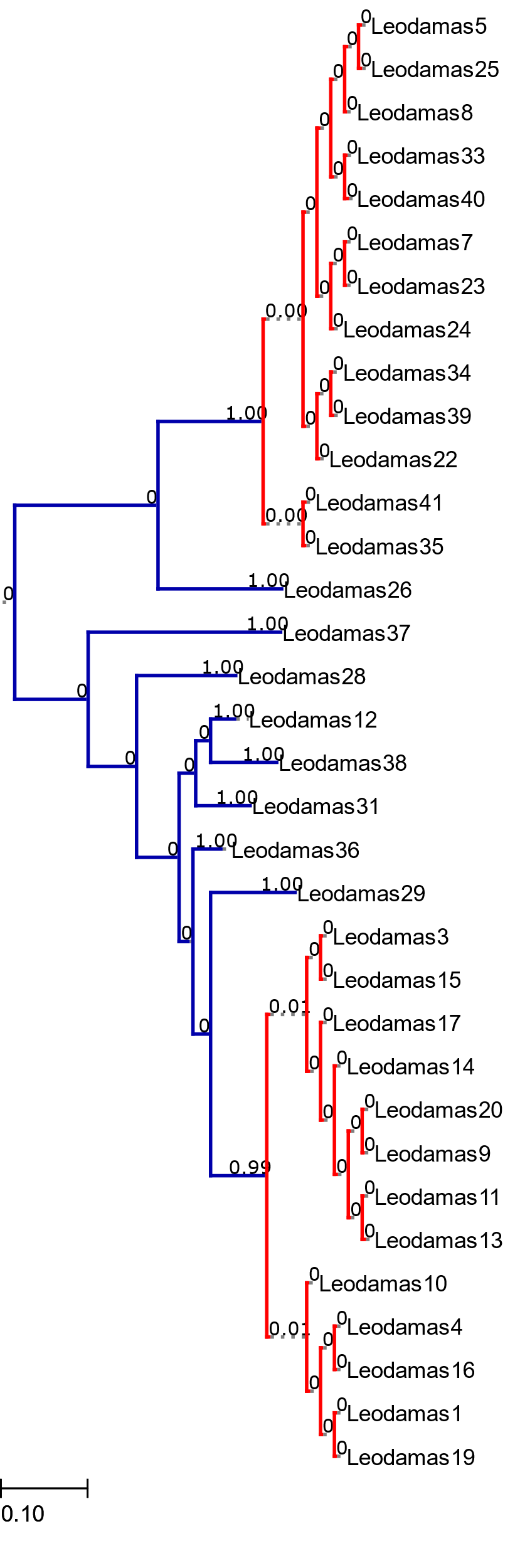


**Maximum Likelihood partition**

Lineage 2 (support = 1.000)

Leodamas37

*Leodamas gracilis* (support = 0.999)

Leodamas5,Leodamas25,Leodamas8,Leodamas33,Leodamas40,Leodamas7,Leodamas23,Leodamas24,Leodamas34,Leodamas39,Leodamas22,Leodamas41,Leodamas35

Lineage 4 (support = 1.000)

Leodamas26

*Leodamas chevalieri* (support = 1.000)

Leodamas28

Lineage 8 (support = 1.000)

Leodamas29

*Leodamas edgari* sp. nov. (support = 0.990)

Leodamas3,Leodamas15,Leodamas17,Leodamas14,Leodamas20,Leodamas9,Leodamas11,Leodamas13,Leodamas10,Leodamas4,Leodamas16,Leodamas1,Leodamas19

Lineage 10 (support = 1.000)

Leodamas36

Lineage 13 (support = 1.000)

Leodamas12

Lineage 14 (support = 1.000)

Leodamas38

Lineage 12 (support = 1.000)

Leodamas31

**Results based on the Bayesian inference reconstruction**

**Bayesian inference tree**


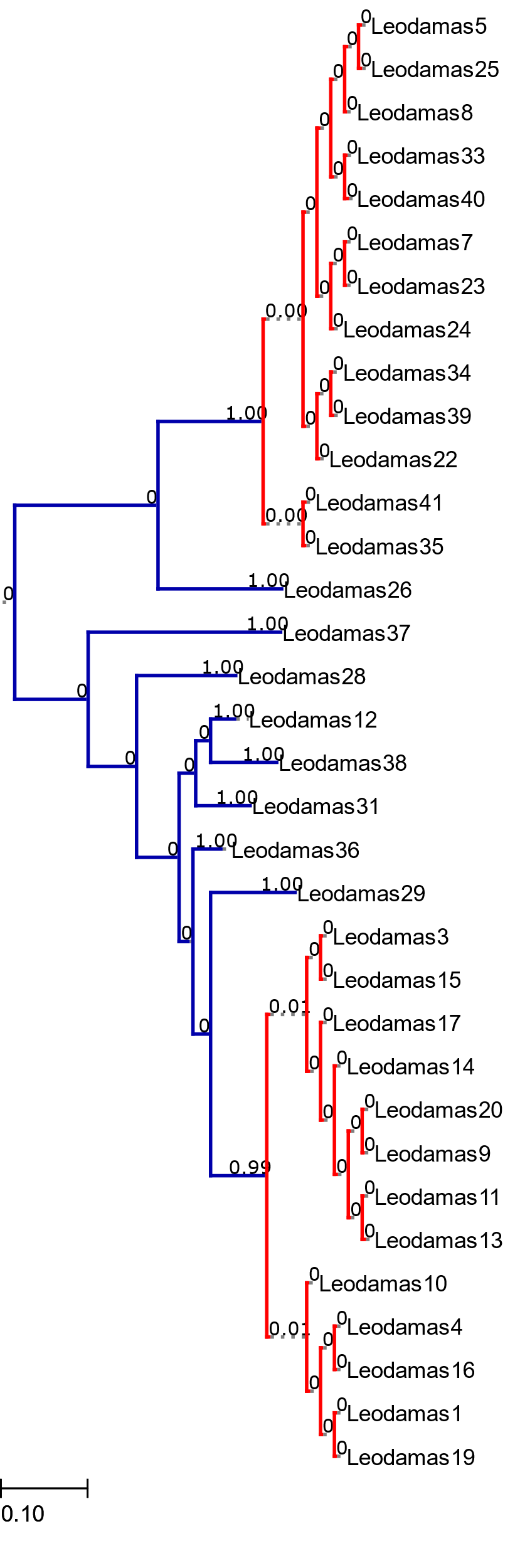


**Most supported partition found by simple heuristic search**

Lineage 2 (support = 1.000)

Leodamas37

*Leodamas gracilis* (support = 0.999)

Leodamas5,Leodamas25,Leodamas8,Leodamas33,Leodamas40,Leodamas7,Leodamas23,Leodamas24,Leodamas34,Leodamas39,Leodamas22,Leodamas41,Leodamas35

Lineage 4 (support = 1.000)

Leodamas26

*Leodamas chevalieri* (support = 1.000)

Leodamas28

Lineage 8 (support = 1.000)

Leodamas29

*Leodamas edgari* sp. nov. (support = 0.990)

Leodamas3,Leodamas15,Leodamas17,Leodamas14,Leodamas20,Leodamas9,Leodamas11,Leodamas13,Leodamas10,Leodamas4,Leodamas16,Leodamas1,Leodamas19

Lineage 10 (support = 1.000)

Leodamas36

Lineage 13 (support = 1.000)

Leodamas12

Lineage 14 (support = 1.000)

Leodamas38

Lineage 12 (support = 1.000)

Leodamas31

**File S2.** ASAP species delimitation. A. COI. B. ITS2.

**A. Species delimitation – COI fragment: ASAP results**

**Partition 1**

Score: 2

Proba: 1.160859e-03

Nb groups:13 (without recursion: 12)

------------------------------------------------------------

Lineage 2[ 1 ] n: 1 ;id: Leodamas37

Lineage 3[ 2 ] n: 1 ;id: Leodamas_tribulosus

Lineage 1[ 3 ] n: 1 ;id: Leodamas_sp

Lineage 11[ 4 ] n: 1 ;id: Leodamas_rubra

*Leodamas edgari* sp. nov.[ 5 ] n: 15 ;id: Leodamas1 Leodamas3 Leodamas4 Leodamas9 Leodamas11 Leodamas13 Leodamas14 Leodamas16 Leodamas6 Leodamas15 Leodamas19 Leodamas20 Leodamas17 Leodamas18 Leodamas10

Lineage 13[ 6 ] n: 1 ;id: Leodamas12

Lineage 14[ 7 ] n: 1 ;id: Leodamas38

Lineage 10[ 8 ] n: 1 ;id: Leodamas36

Lineage 7[ 9 ] n: 1 ;id: Leodamas32

Subset[ 10 ] n: 2 ;id: Leodamas29 Leodamas30

*Leodamas gracilis*[ 11 ] n: 14 ;id: Leodamas5 Leodamas21 Leodamas7 Leodamas34 Leodamas8 Leodamas33 Leodamas41 Leodamas22 Leodamas35 Leodamas40 Leodamas39 Leodamas23 Leodamas24 Leodamas25

Lineage 4[ 12 ] n: 2 ;id: Leodamas26 Leodamas27

*Leodamas chevalieri*[ 13 ] n: 1 ;id: Leodamas28

**Partition 2**

Score: 2.5

Proba: 9.999900e-06

Nb groups:12 (without recursion: 10)

------------------------------------------------------------

Subset[ 1 ] n: 2 ;id: Leodamas37 Leodamas_tribulosus

Lineage 1[ 2 ] n: 1 ;id: Leodamas_sp

Lineage 11[ 3 ] n: 1 ;id: Leodamas_rubra

*Leodamas edgari* sp. nov.[ 4 ] n: 15 ;id: Leodamas1 Leodamas3 Leodamas4 Leodamas9 Leodamas11 Leodamas13 Leodamas14 Leodamas16 Leodamas6 Leodamas15 Leodamas19 Leodamas20 Leodamas17 Leodamas18 Leodamas10

Lineage 13[ 5 ] n: 1 ;id: Leodamas12

Lineage 14[ 6 ] n: 1 ;id: Leodamas38

Lineage 10[ 7 ] n: 1 ;id: Leodamas36

Lineage 7[ 8 ] n: 1 ;id: Leodamas32

Subset[ 9 ] n: 2 ;id: Leodamas29 Leodamas30

*Leodamas edgari* sp. nov.[ 10 ] n: 14 ;id: Leodamas5 Leodamas21 Leodamas7 Leodamas34 Leodamas8 Leodamas33 Leodamas41 Leodamas22 Leodamas35 Leodamas40 Leodamas39 Leodamas23 Leodamas24 Leodamas25

Lineage 4[ 11 ] n: 2 ;id: Leodamas26 Leodamas27

Subset[ 12 ] n: 1 ;id: Leodamas28

**B. Species delimitation – ITS2 fragment: ASAP results**

**Partition 1**

Score: 2

Proba: 9.999900e-06

Nb groups:9 (without recursion: 8)

------------------------------------------------------------

*Leodamas edgari* sp. nov.[ 1 ] n: 13 ;id: Leodamas1 Leodamas19 Leodamas4 Leodamas16 Leodamas10 Leodamas11 Leodamas3 Leodamas15 Leodamas17 Leodamas14 Leodamas20 Leodamas9 Leodamas13

Subset[ 2 ] n: 2 ;id: Leodamas12 Leodamas36

Lineage 12[ 3 ] n: 1 ;id: Leodamas31

Lineage 8[ 4 ] n: 1 ;id: Leodamas29

*Leodamas chevalieri*[ 5 ] n: 1 ;id: Leodamas28

Lineage 14[ 6 ] n: 1 ;id: Leodamas38

Lineage 2[ 7 ] n: 1 ;id: Leodamas37

*Leodamas gracilis*[ 8 ] n: 13 ;id: Leodamas5 Leodamas7 Leodamas23 Leodamas8 Leodamas33 Leodamas24 Leodamas34 Leodamas39 Leodamas22 Leodamas41 Leodamas35 Leodamas40 Leodamas25

Lineage 4[ 9 ] n: 1 ;id: Leodamas26

**Partition 2**

Score: 2

Proba: 9.999900e-06

Nb groups:4 (without recursion: 3)

------------------------------------------------------------

Subset[ 1 ] n: 19 ;id: Leodamas1 Leodamas19 Leodamas4 Leodamas16 Leodamas10 Leodamas11 Leodamas3 Leodamas15 Leodamas17 Leodamas14 Leodamas20 Leodamas9 Leodamas13 Leodamas12 Leodamas36 Leodamas31 Leodamas38 Leodamas29 Leodamas28

Lineage 2[ 2 ] n: 1 ;id: Leodamas37

*Leodamas gracilis*[ 3 ] n: 13 ;id: Leodamas5 Leodamas7 Leodamas23 Leodamas8 Leodamas33 Leodamas24 Leodamas34 Leodamas39 Leodamas22 Leodamas41 Leodamas35 Leodamas40 Leodamas25

Lineage 4[ 4 ] n: 1 ;id: Leodamas26
